# Subtractive proteomics to identify novel drug targets and reverse vaccinology for the development of chimeric vaccine against *Acinetobacter baumannii*

**DOI:** 10.1038/s41598-018-26689-7

**Published:** 2018-06-13

**Authors:** Vandana Solanki, Vishvanath Tiwari

**Affiliations:** Department of Biochemistry, Central University of Rajasthan, Bandarsindri, Ajmer, 305817 India

## Abstract

The emergence of drug-resistant *Acinetobacter baumannii* is the global health problem associated with high mortality and morbidity. Therefore it is high time to find a suitable therapeutics for this pathogen. In the present study, subtractive proteomics along with reverse vaccinology approaches were used to predict suitable therapeutics against *A. baumannii*. Using subtractive proteomics, we have identified promiscuous antigenic membrane proteins that contain the virulence factors, resistance factors and essentiality factor for this pathogenic bacteria. Selected promiscuous targeted membrane proteins were used for the design of chimeric-subunit vaccine with the help of reverse vaccinology. Available best tools and servers were used for the identification of MHC class I, II and B cell epitopes. All selected epitopes were further shortlisted computationally to know their immunogenicity, antigenicity, allergenicity, conservancy and toxicity potentials. Immunogenic predicted promiscuous peptides used for the development of chimeric subunit vaccine with immune-modulating adjuvants, linkers, and PADRE (Pan HLA-DR epitopes) amino acid sequence. Designed vaccine construct V4 also interact with the MHC, and TLR4/MD2 complex as confirm by docking and molecular dynamics simulation studies. Therefore designed vaccine construct V4 can be developed to control the host-pathogen interaction or infection caused by *A. baumannii*.

## Introduction

*Acinetobacter baumannii*, an ESKAPE pathogen, has gained the attention of medical fraternity worldwide due to its nosocomial infection in hospital setup mainly ICUs and emergence of multi-drug resistance mechanism in it^1–6^. *A. baumannii* have developed MDR, XDR, and PDR strain^7^. Bacterial pathogen interacts with the host and has also developed several strategies to evade the host immune system. Therefore, it is high time to develop suitable therapeutics or vaccine against the *A. baumannii*.

Previous studies reveal that outer membrane proteins OmpA, biofilm-associated protein, poly-N-acetyl-β-(1–6)-glucosamine^8^, trimeric autotransporter protein, K1 capsular polysaccharide, outer membrane vesicles (OMV) and formalin-inactivated whole cells could serve as vaccine candidates and provide partial immunity against lethal doses in various mouse models^9^. Recently, it is shown that subtractive genomics and reverse vaccinology as a powerful tool to identify drug target and vaccine candidates. Although both these approaches have been used separately to design the novel drugs and vaccines against Gram-negative bacteria^10,11^. Currently, vast information about genomes and proteomes of *A. baumannii* strains are available and promising vaccine candidates, or novel proteins can be identified using the computational tools^12^. Subtractive genomics approach subtracts pathogen genes that are required the survival of pathogen but not present in the host^13^. This is important to find druggable protein, which may be considered for the therapeutics development. The selected druggable proteins may be used for the development of the chimeric-subunit vaccine or multi-subunit vaccine that appears as a very promising and effective treatment option to control the diseases caused by this pathogen^14^. Once shortlisted, these candidates can be cloned and over-expressed in *E. coli* and purified by affinity chromatography. Their immunogenicity can be validated *in-vivo* in suitable animal models.

In addition to essential proteins, virulence factors and resistant determinants also mediates bacterial attachments that may contribute to the pathogenicity of the bacterium^15^. Cytoplasmic proteins are usually considered for small molecule drug development while membrane or secreted proteins are considered for vaccine development^16^. Therefore, the present study aims to identify the druggable essential and virulence proteins from the different strains of *A. baumannii*. The identified proteins will be used for selection of promiscuous immunogenic non-allergenic epitopes. The selected epitopes will be used in the development of chimeric subunit vaccine. The designed chimeric-subunit vaccine can be developed to cure the infection caused by the *A. baumannii*.

## Methods

### Data collection of proteome

In the present study, list of all available strains of *A. baumannii* have been downloaded from UNIPORT server. *Acinetobacter* species have more than 32 genospecies, which include major four genospecies like *A. baumannii, A. calcoaceticus, A. pittil*, and *A. nosocomialis*. *A. baumannii* was filtered out from the list and used for further analysis. Bacterial proteome redundancy is a barrier to the effective use of the dataset for multiple reasons, removing redundant sequences is desirable to avoid highly repetitive search results for queries that closely match with an over-represented sequence. Hence, all the searched strains from the UNIPORT proteome were separated according to their redundancy and non-redundancy. All the selected 52 proteomes (including reference proteome) were downloaded from UNIPORT database, and 51 proteomes were subjected to BLASTp against reference strain (SDF). The obtained shared proteins were used for further analysis. The proteins having sequence length less than 100 amino acids were also considered^17^.

### Data collection of the genome and phylogenetic analysis

Genome data of selected 52 strains of *A. baumannii* were obtained from NCBI. INSDC (International Nucleotide Sequence Database Collaboration) numbers were used for the complete genome where as WGS number used for the draft sequence. The whole genome DNA sequence was searched for rRNA sequences using RNAmmer^18^. One 16S rRNA gene was randomly sampled per strain because there are only small sequence differences among 16S rRNA genes within the same genome and the same species. Phylogeny tree was constructed using MEGA6 where 16S rRNA gene sequences from the genome of the all *A. baumannii* strains were used^19^. The alignment program ClustalW was used for multiple sequence alignment of the sequences. From the alignment, a distance neighborhood joining tree was constructed, using 1000 bootstraps to find the best fitting distance tree^20^.

### CD-HIT analysis

**Figure 1:**
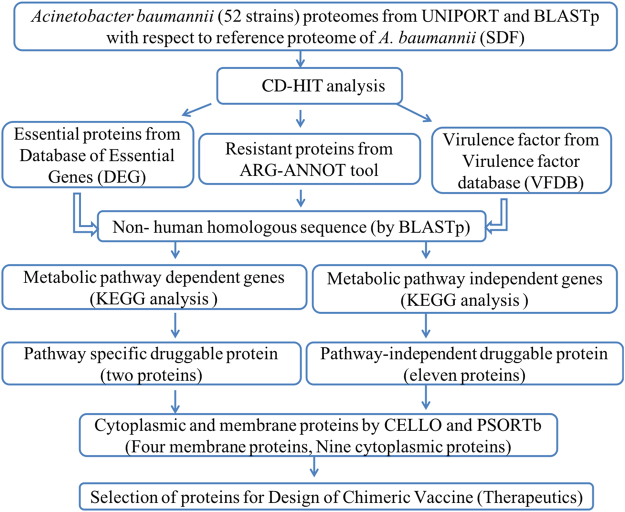
Illustration of predefined comparative and subtractive proteomics systemic workflow.

Subtractive analysis (Fig. 1) of proteins were performed using CD-HIT to identify the duplicate proteins by clustering techniques. Sequence identity cut-off was kept at 0.6 (60% identity) as sequence having identity >60% similar/related structure and functions^21^. Global sequence identity algorithm was selected for the alignment of the amino acids. The bandwidth of 20 amino acids and default parameters for alignment coverage were selected.

### Screening of essential proteins

The database of essential genes (DEG) (http://tubic.tju.edu.cn/deg/) includes essential protein-coding genes determined by genome-wide gene essentiality analysis. DEG consists of experimentally identified 22,343 essential protein-coding genes and proteins, 646 non-coding RNAs, promoters, regulatory sequences, and replication origins from 31 prokaryotes and 10 eukaryotes^22^. The queried proteins having homologous hit in DEG are likely to be essential. BLASTp search was performed for the proteome of *A. baumannii* against DEG bacterial proteins with cut-off parameters of 1e^−04^ E-value, bit score of 100, BLOSUM62 matrix and gapped alignment mode were selected to screen out the essential proteins.

### Analysis of virulence factors (VF’s)

Virulence factors help bacteria to modulate or degrade host defense mechanism with the help of adhesion, colonization, and invasion resulted cause the disease. VFDB, a database consists of four categories of VFs namely offensive, defensive, non-specific and virulence-associated regulated proteins from 25 pathogenic bacteria were used in the present study^23^. The proteome of *A. baumannii* was subjected to BLASTp search against the database of protein sequences from VFDB core dataset (R1) with default hit with cut-off bit score >100, and E-value was 0.0001.

### Analysis of resistance proteins

ARG-ANNOT (Antibiotic Resistance Gene-ANNOTation) is a bioinformatics tool that detects existing and putative new antibiotic resistance (AR) genes or proteins in bacterial genomes or proteome. ARG-ANNOT contains 1689 antibiotic resistant protein sequences from various classes including aminoglycosides, beta-lactamases, fosfomycin, fluoroquinolones, glycopeptides, phenicols, rifampicin, sulfonamides, tetracyclines, and trimethoprim. A local BLAST program was run for the proteome of *A. baumannii* in Bio-edit software against antibiotic resistant sequences in ARG-ANNOT with cut-off E-value of 1e^−04^ ^24^.

### Selection of non-homologous proteins to host

BLASTp search of the comprised list from the above three independent searches were performed against non-redundant protein sequence (nr) database of the host *Homo sapiens* (taxid: 9606). The comparison of proteins with human host protein finds the non-hit proteins lists that denote non-human homologous proteins of the pathogens. This will help to design the pathogen specific therapeutics drugs^25^.

### Pathogen-specific pathways

KEGG (Kyoto encyclopedia of gene and genome) is a pathway database^26^ that is to find metabolic pathways of non-homologous proteins of *A. baumannii*. Similarly, host metabolic pathways were also enlisted along with their K number. The enlisted host and pathogen metabolic pathways were manually compared to identify the pathways that present only in the pathogen but not in the human host. The list of proteins that plays a role in unique pathways was enlisted. The proteins were also separated according to their role only in pathogen-specific unique pathways and common pathways present in both pathogen and host.

### Druggability analysis

Druggability analysis of the short-listed proteins searched against all drug targets present in the DrugBank database^27^. The drug targets with bit score >100 and E value < 0.005 were considered as a potential drug target. Biological location of druggable proteins was classified based on the consensus location predicted using various online servers like PSORTb^28^ and CELLO v2.5 (http://cello.life.nctu.edu.tw). The low molecular weights (100–110 kDa) target proteins increase the accessibility value of the targets proteins.

### Prediction of antigenic protein

**Figure 2:**
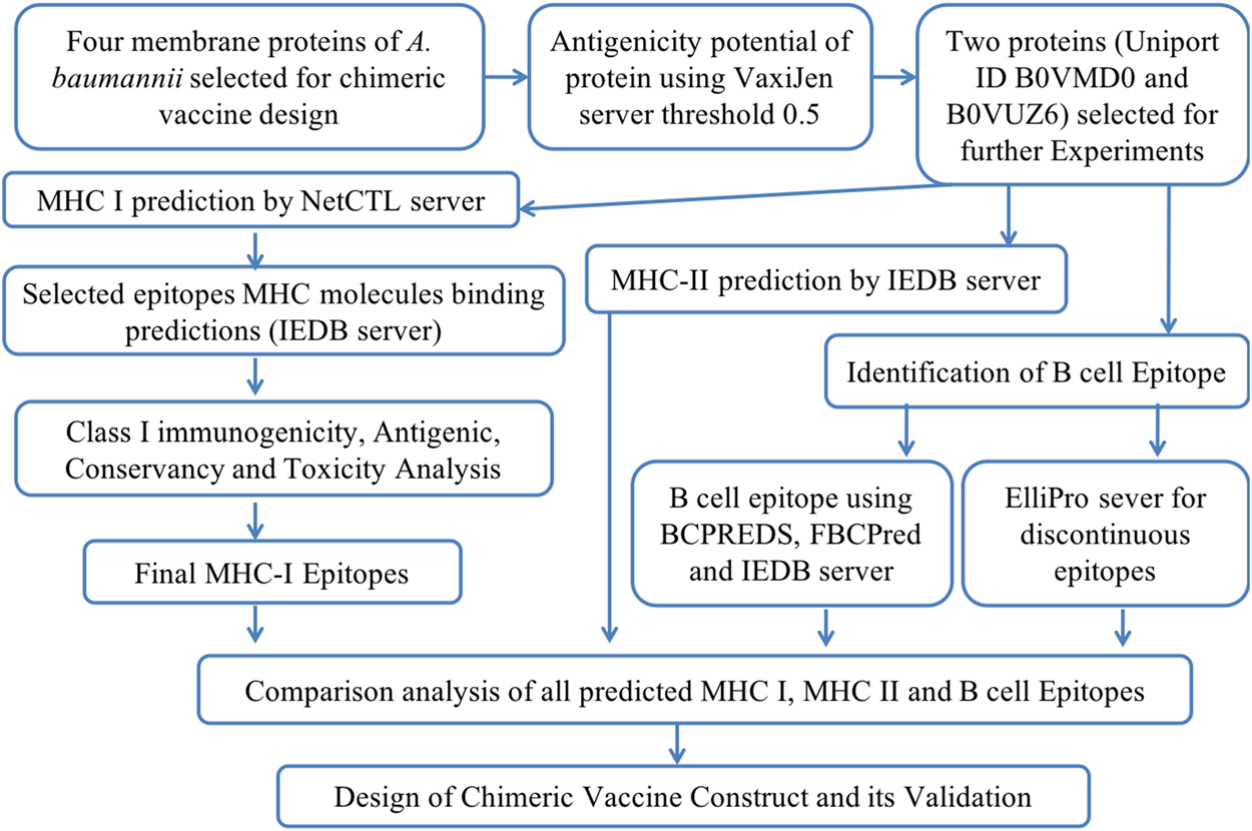
Illustration of reverse vaccine devolvement workflow.

From subtractive proteomics approach, outer membrane proteins were selected that contained the potential for vaccine development. These protein targets were used for the bioinformatics study (Fig. 2) to identify the potent epitopes that can enhance the immune response. Antigenic property of all the selected proteins was determined using VaxiJen web server^29^. A threshold of 0.5 was considered as the potent antigenicity. Identified potent epitope containing proteins were used for afterward analysis.

### Protein-protein interaction network analysis

Protein interaction carried to find out the most potential metabolic functional associations among all identified proteins through protein interaction database STRING (http://string-db.org). The STRING database aims to provide a critical assessment and integration of protein-protein interactions, including direct as well as indirect associations.

### T cell MHC Class I epitope prediction

Predictions of MHC class I epitopes were made by the NetCTL server^30^ for the selected proteins. In this server, epitopes were chosen on the basis of the high overall combinatorial score for each peptide’s intrinsic potential. In integrating with several predictions methods such as proteasomal cleavage, TAP transporter associated efficiency with antigen processing, and MHC-I affinity predictor, scores of all three methods were merged together and achieved combined score for all the predicted epitopes. Prediction threshold value 0.75 was set for epitope identification.

At the last stage, 40 T-cell epitopes were subjected to MHC-I binding prediction, using the immune epitope database analysis resource (IEDB AR)^31^. MHC molecules represent the antigenic peptides which are recognized by the T-cells. IEDB recommended as a default prediction method and uses the consensus method consisting of ANN^32^, SMM^33^, and CombLib^34^ and NetMHCpan^35^. Identified T-cell epitopes with HLA alleles were selected by IC_50_ values and percentile rank. Lower the percentile rank the higher the interaction shown between the peptide and MHC molecules. IC_50_ values divided in three different categories: high binding affinity, IC_50_ value < 50 nM; intermediate binding affinity, IC_50_ value < 500 nM; and low binding affinity, IC_50_ value < 5000 nM^36^.

### Class I immunogenicity prediction

Epitope/MHC complex should have the ability to evoke an immune response. Hence we have used MHC I immunogenicity prediction tool using IEDB server^37^. Default parameters were selected to perform the immunogenicity prediction. The epitopes which, immunogenicity prediction gained the positive value were selected for further analysis. (http://tools.immuneepitope.org/immunogenicity/).

### Antigenic, Conservancy and Toxicity Analysis

All the promiscuous epitopes obtained from immunogenicity tool were analysed using VaxiJen version 2.0 server for their antigenic properties at a threshold value of 0.5. This server depends on Auto Cross Covariance (ACC) transformation, and alignment-independent predicted antigenic epitopes by their physicochemical behaviour. To assess the epitopes conservancy level within genotype sequences, IEDB conservancy analysis^38^ was used. The sequence identity parameters were set to default. This analysis aims to calculate the degree of conservancy of epitopes within a given protein sequence^39^. ToxinPred (http://www.imtech.res.in/raghava/toxinpred/index.html) online server tool analysed physicochemical properties of epitopes to predict the toxicity level. It confirms that the specific immune responses which are induced in the host cell will target only the bacteria rather than host tissue^40^. The parameters were set to default.

### T cell MHC Class II epitope prediction

T-cell epitopes binding to MHC Class II molecules were predicted using IEDB-AR server. The T-cell epitopes were computed using the consensus method^41,42^. The consensus prediction approach uses a combination of both stabilization matrix alignment method and average relative binding matrix method.

### Cluster analysis of the MHC restricted Alleles

MHCcluster v2.0 server^43^ identified the cluster of MHC restricted allele with appropriate peptides to further strengthen our prediction. This is the additional crosscheck of the predicted MHC restricted allele analysis from the IEDB analysis resources. The output from this server is a graphical tree and static heat map for describing the peptides and HLAs functional relationship.

### B-cell epitope prediction

Prediction of linear B-cell epitopes for proteins was achieved by using online server BCPREDS, i.e., B-cell Epitope Prediction Server (http://ailab.ist.psu.edu/bcpred/predict.html)^44^ and FBCPred^45^ server. BCPred based on five different kernel methods having fivefold cross-validation by SVM. FBCPred utilizes subsequent kernel for the prediction of linear length B-cell epitopes. The cut-off score of BCPreds is >0.8 for prediction of linear B-cell epitopes^11^. The goal of B-cell epitope prediction was used to determine the antigen recognized by B lymphocytes and initiate humoral immunity. IEDB B-cell epitope prediction server predicted the linear epitopes using biochemical properties such as amino acid composition, hydrophobicity, hydrophilicity, surface accessibility, and secondary structure. This server is consists of BepiPred linear epitope prediction^46^, Karplus-Schulz flexibility prediction^47^, Chou-Fasman beta-turn prediction^48^, Kolaskar Tongaonkar antigenicity^49^, Emini surface accessibility prediction^50^, and Parker hydrophilicity prediction^51^. ElliPro server^52^ of IEDB identify the linear and conformational epitopes of B-cell. This server predicted epitopes with a score, defined as a Protrusion Index (PI) value averaged over epitope residues. In the method, the protein’s 3D shape is approximated by some ellipsoids. Residues with larger scores are associated with greater solvent accessibility. Discontinuous epitopes are also defined based on PI values.

### Construction of model vaccine

To construct the novel vaccine with low toxicity, allergenicity, and highly immunogenicity, we have analysed the different combination of sequence constructs. During this vaccine construction, firstly, sequence 62–106, 359–419 of B0VMD0 (uniport ID), and epitope sequence 688–787, 79–139, 366–382 of B0VUZ6 protein were joined with the help of amino acid linkers. Secondly, epitopes 162–204, 231–336 of B0VMD0 protein, and epitope 174–248, 636–659 of B0VUZ6 proteins were added with the help of linkers. For enhancing the immunogenicity of these two sequence constructs were added with the four different adjuvants L7/L12 ribosomal protein, beta-defensin, HBHA protein (*M. tuberculosis*, accession no. AGV15514.1), and HBHA conserved sequence^53^ respectively. PADRE peptide sequences were also incorporated along with the adjuvants. PADRE peptide induced CD4^+^ T-cells that improve efficacy and potency of peptide vaccine^54^. Adjuvant HBHA and L7/L12 ribosomal protein are agonists to the TLR4/MD2 complex while beta-defensin adjuvant is agonist to TLR1, TLR2, and TLR4. The TLR’s interaction polarizes CTL responses which have a robust immuno-stimulatory effect^53^. HEYGAEALERAG and GGGS linkers were conjugated with HTL, CTL and B epitopes, whereas adjuvants sequences were linked with the help of EAAAK linkers at both N- and C-terminus. Yang *et al*. proved that ‘GGGS linkers superior to ‘AAY’ as a ‘linkers’ of epitope-based vaccines^55^. The fused vaccine constructs were used for further analysis.

### Allergenicity, Antigenicity and solubility evaluation

Allergenicity was predicted using AlgPred program^56^. AlgPred uses all these parameters (IgE epitope + ARPs BLAST + MAST + SVM) combined to predict the allergenicity of peptide with the accuracy of 85%. A threshold score of prediction has been considered to be −0.40. Prediction score less than threshold value point towards the non-allergic behaviour of the peptide. For identification of vaccine antigenicity, ANTIGENpro program^57^ (accuracy of 76%) and VaxiJen v2.0 program were used. SOLpro program^58^, predicts the propensity of vaccine to be soluble upon over-expression in *E. coli*. Two-stage SVM architecture was employed based on multiple representations of the primary amino acid sequence. The final result was summarizing the predictions with overall accuracy of 74% at corresponding probability (≥0.5).

### Secondary structure prediction

The PSIPRED v3.3 program was used^59^ for the secondary structure analysis (http://bioinf.cs.ucl.ac.uk/psipred/). Psi-Blast was performed to identify and select the related sequences showing significant homology to the vaccine protein. An average Q-3 score of PSIPRED 3.2 server is 81.6%.

### Prediction of various physicochemical properties

Vaccine sequences were functionally characterized using Expasy ProtParam server (http://expasy.org/cgi-bin/protpraram). This server is widely used for determining the physicochemical characteristics like the number of amino acids, molecular weight, PI values, hydropathicity GRAVY values, instability index, aliphatic index, and estimated half-life of the protein of a generated protein model. It can compute various physicochemical properties on the basis of pK values of different amino acids. Instability index of protein predicts whether it is stable or unstable. Instability index value is <40 for stable protein and >40 for unstable protein. The volume occupied by the aliphatic side chains is known as the aliphatic index of protein. Grand average of hydropathicity was calculated by the sum of hydropathicity obtained for all of the amino acid residues divided by the total number of amino acid residues present in the protein.

### Molecular Docking and Molecular Dynamics Simulation

With the help of the Phre2 online tool, we have made models of all the four vaccine constructs. PDB ID of all HLA alleles were downloaded from protein data bank, RCSB. Molecular docking of the final four vaccine constructs were performed with six different HLA alleles i.e. 1A6A(HLA-DR B1*03:01), 3C5J(HLA-DR B3*02:02), 1H15(HLA-DR B5*01:01), 2FSE(HLA-DR B1*01:01), 2Q6W(HLA-DR B3*01:01), and 2SEB(HLA-DRB1*04:01) were performed using PatchDock to show HLA-peptide interactions. For more, refinement and re-scoring of rigid body molecular docking score, FireDock (Fast Interaction Refinement in Molecular Docking) server was used. It gives best 10 solutions for final refinement. The refined models were based on the binding score and global binding energy. Similarly, docking of vaccine construct (V4) with TLR 4/MD2 complex (PDB ID 2Z65) was performed by PatchDock server. The generated top 10 models were refined and re-scored by FireDock server. The refined candidates were ranked by their respective binding energy. Molecular dynamics simulation of V4-TLR4 complex was performed using Gromacs v5.1.2 as published method^60^.

### Codon optimization of the vaccine construct and *In-silico* cloning

Java Codon Adaptation Tool (JCAT) was performed to adapt the codon usage of vaccine to *E. coli* host strain^61^. Vaccine amino acid sequence was back-translated to DNA, and subsequently adapted for codon usage to *E. coli*. The adaptation was based on Codon Adaptation Index values (CAI), that were computed by employing an algorithm. The rho-independent transcription terminators, prokaryotic ribosome binding sites and cleavage sites of some restriction enzymes were avoided. Moreover, to clone the adapted gene sequence of final vaccine construct in *E. coli* pET28a vector, using Snapgene tool to ensure the vaccine construct expression.

### Data availability

All data generated or analysed during this study are included in this published article and its Supplementary Information files.

### Ethical approval

The present study does not involved human or animal samples.

## Results

### Subtractive proteomics approach shortlisted *A. baumannii* strains

We have downloaded the list of 1753 different strains of *Acinetobacter genospecies* from the UNIPORT. A total of 1578 strains of *Acinetobacter baumannii* were filtered from the lists and used for further analysis. All the 1578 strains of *A. baumannii* were manually separated according to their redundancy and non-redundancy. In this manual comparison, we have made 29 different groups of non-redundant strains that contain the list of redundant strains. Twenty-three strains of *A. baumannii* did not show any redundancy with the other *Acinetobacter* strains. Therefore a total number of 52 (29 + 23) strains were selected for the analysis. Proteome of *A. baumannii* SDF strain was considered as reference proteome that contains most representative and best annotated set of proteins of all *A. baumannii* strains. The proteomes of all 52 *A. baumannii* strains were retrieved from UNIPORT database. With the help of BLASTp, we have found the similar protein present in the all 51 proteomes of *A. baumannii* with respect to reference proteome SDF.

### Phylogenic analysis of selected 52 *A. baumannii* strains showed their inter-relation

INSDC numbers or WGS numbers of all 52 strains of *A. baumannii* were downloaded from NCBI (Supplementary Table ST-1). The results of RNAmmer analysis yielded no rRNA sequences for five genomes (*A. baumannii* 118362, 855125, 940793, ABBL059 and ABBL059). These five genomes would have some unknown base stretches that prevent RNAmmer from identifying rRNA sequences^20^. Genome sequence lack 16S rRNA sequences that might be a result of sequence assembly. Since ribosomal RNA sequences often are repeated sequences, the assembly process might not be able to conclusively place the rRNA in the DNA and might discard the sequences all-together^62^. Distance-based phylogenic analysis measured dissimilarity observe between pairs of sequence alignment. Length of branch represented an amount genetic change of 0.05. The sum of branch length (SBL) value 0.437 was observed (Supplementary Figure SF1) in the analysis of distance based phylogenic tree.

### Identification of essential proteins of *A. baumannii*

From the DEG result, 811 proteins of *A. baumannii* were found to be essential, and rest of them were known as non-essential proteins. These 811 essential proteins are necessary for the survival of *A. baumannii*. Blocking these bacterial proteins will cause the death of the micro-organism, which will make the protein targets more important in drug discovery.

### Identification of virulence factors of *A. baumannii*

Analysis of virulence factors explored the significance of bacteria in various diseases necessitates the search for novel VFs. The VFDB result showed that 339 proteins were found to be associated with virulence of *A. baumannii*. These proteins may also be considered as a very important target to inhibit the pathogenesis of *A. baumannii*.

### Identification of resistant determinant in *A. baumannii*

Protein involved in the resistance and drug efflux proteins could be act as potential therapeutic targets to inhibit the resistant strain of *A. baumannii*. We have obtained 10 antibiotic resistant proteins with cut-off E-value of 1e^−04^. These ten proteins are involved in the degradation of antibiotics and efflux of the antibiotics.

### Identification of non-human homologous proteins in *A. baumannii*

The manual comparative analysis of the proteins showed that 35 proteins contained the both essential and virulence property. The search of the non-redundant proteins of essential, virulence and resistance datasets is necessary to evaluate homology of these protein with human, to find non-human homologous proteins in *A. baumannii* that further enhances the specificity of the designed drug or vaccine. We have found 174 VF’s protein, 347 essential protein and 5 antibiotic resistance protein that are non-human homologous protein. Remaining 165 VF’s, 459 essential and 3 resistance proteins showed similarity with human proteins, therefore, filter out from further use. Detection of non-human homologous proteins to find out the proteins were likely to lead to drug development exclusively interact with proteins of *A. baumannii*.

### Involvement of selected *A. baumannii* proteins in the unique and common pathway

We have done the manual comparison between the enlisted metabolic pathways of *A. baumannii* and pathways of human (present in KEGG Database). The result showed that 33 unique pathways (Supplementary Table 2) are present in the *A. baumannii* pathogen and rest 104 pathways were common in both bacteria and host. These 33 unique pathways were found to possess 126 proteins. Out of 126 proteins, 34 proteins play a role in unique *A. baumannii* specific pathways while 92 proteins have a role both in unique and common pathways of pathogen and human. Therefore, 34 proteins have been selected for further analysis. The 358 proteins are not present in any metabolic pathway are considered as pathway independent protein analysis.

### Identification of putative drug targets for therapeutics development

#### Pathway dependent protein target

Druggability analysis showed that only two proteins of unique pathway were druggable in nature that include penicillin-binding protein 1B protein (that have role in peptidoglycan biosynthesis) and channel-tunnel spanning the outer membrane and periplasm segregation of daughter chromosomes protein (that have role in bacterial secretion system, two-component system, beta-lactam resistance and CAMP resistance pathway). The detailed drug name, drugbank ID, E-value and bit score value of the target proteins are mentioned in Table 1. These proteins uniquely play a role in different pathways of *A. baumannii* thus can be targeted for vaccine or drug discovery.

**Table 1:** Novel druggable targets involved in different metabolic dependent or independent pathways.

S.No.	Protein Name	Uniport ID	Drug bank ID	Drug name	E value	Bit score	Query length
**Metabolic pathway dependent druggable proteins**							
1	Penicillin-binding protein 1B OS	B0VUZ6	DB01598, DB01329, DB01332, DB01327, DB01331, DB01328, DB01415, DB00430, DB00438, DB00274	Imipenem, Cefoperazone, Ceftizoxime, Cefazolin, Cefoxitin, Cefonicid, Ceftibuten, Cefpiramide, Ceftazidime, Cefmetazole	4.23297e-147	450.669	756
2	Channel-tunnel spanning the outer membrane and periplasm segregation of daughter chromosomes	B0VMD0	DB03350	Cobalt Hexammine Ion	1.65352E-39	146.362	438
**Metabolic pathway independent druggable proteins**							
1	DNA gyrase subunit A	B0VT39	DB00817, DB11943	Rosoxacin, Delafloxacin	4.1128E-130	406.757	720
2	Putative UDP-glucose 6-dehydrogenase (Ugd)	B0VMS1	DB00157, DB09130	NADH, copper	9.79852E-31	120.939	305
3	Bifunctional purine biosynthesis protein PurH	B0VLK7	DB01700, DB01972, DB02309, DB03442, DB04057, DB00116, DB00642, DB00563	AICA ribonucleotide, Guanosine-5′-Monophosphate, 5–Monophosphate-9-Beta-D-Ribofuranosyl Xanthine, 2-[5-Hydroxy-3-Methyl-1-(2-Methyl-4-Sulfo-Phenyl)−1h-Pyrazol-4-Ylazo]-4-Sulfo-Benzoic Acid, Beta-Dadf, Msa, Multisubstrate Adduct Inhibitor, Tetrahydrofolic acid, Pemetrexed, Methotrexate	1.01028E-90	287.345	507
4	Ferredoxin–NADP + reductase	B0VUY5	DB03147	Flavin adenine dinucleotide	4.86153E-40	136.732	237
5	Pyridoxine 5′-phosphate synthase	B0VTM9	DB02209, DB02496, DB02515	Pyridoxine phosphate, 1-Deoxy-D-xylulose 5-phosphate, 3-Phosphoglycerol	5.21185E-86	253.447	231
6	Malonyl CoA-acyl carrier protein transacylase	B0VTY4	DB07344	3,6,9,12,15-PENTAOXAHEPTADECAN-1-OL	1.30075E-31	119.783	309
7	Dihydropteroate synthase	B0VSM6	DB00576, DB01298, DB00263, DB00634, DB00259, DB01015, DB01581, DB01582, DB06729	Sulfamethizole, Sulfacytine, Sulfisoxazole, Sulfacetamide, Sulfanilamide, Sulfamethoxazole, Sulfamerazine, Sulfamethazine, Sulfaphenazole	2.42841E-79	239.58	267
8	Putative D-ala-D-ala carboxypeptidase, penicillin-binding protein	B0VTR5	DB04647, DB01329, DB01331, DB00274	BOC-GAMMA-D-GLU-L-LYS(CBZ)-D-BOROALA, Cefoperazone, Cefoxitin, Cefmetazole	6.22477E-44	156.377	401
9	3-oxoacyl-[acyl-carrier-protein] reductase	B0VTY3	DB03461	2′-Monophosphoadenosine 5′-Diphosphoribose	2.14867E-26	100.908	233
10	Putative Oxidoreductase, short chain dehydrogenase /reductase family	B0VT18	DB00157	NADH	7.13808E-28	106.301	186
11	Putative acetyl-coA synthetase/AMP-(Fatty) acid ligase	B0VNG2	DB00131, DB00171, DB09395	Adenosine monophosphate, ATP, Sodium acetate	2.32E-70	236.884	568

#### Pathway independent protein target

The 358 metabolic pathways independent proteins of *A. baumannii* screened for druggability test. The result showed the 11 druggable target that includes DNA gyrase subunit A, bifunctional purine biosynthesis protein PurH, ferredoxin-NADP reductase, malonyl coA-acyl carrier protein transacylase, dihydropteroate synthase, pyridoxine 5′-phosphate synthase, putative UDP-glucose 6-dehydrogenase (Ugd), putative acetyl-coA synthetase/AMP-(Fatty) acid ligase, and 3-oxoacyl-(acyl-carrier-protein) reductase proteins were present in the cytoplasm whereas putative D-ala-D-ala-carboxypeptidase, penicillin-binding protein, putative oxidoreductase, and short chain dehydrogenase/reductase family present at the membrane choose as druggable targets on the basis of E-value and the bit score value. The detailed drug name, drugbank ID, the E value and bit score value mentioned in Table 1.

### Identification of subcellular localization and prioritization of selected target proteins

Although non-homologous proteins of the pathogen represent potential targets for therapeutic and vaccine candidates, prioritization and more filtration of the identified proteins could help to minimize the time, labor and resources for developing the therapeutic agent and optimize the success of getting the best drug and/or vaccine against the pathogen. Hence, additional parameters that determine the suitability of a drug and vaccine target were used to characterize the identified target proteins. The different characterizations of proteins like, sub-cellular localization by CELLO and PSORTb, molecular weights by Swiss-Prot database, and TMHMM which identify trans-membrane proteins. Prediction of sub-cellular localization of druggable targets using various online search tools showed that in the pathway dependent proteins both proteins presented in the outer membrane. In the pathway independent proteins, 9 proteins presented in the cytoplasm and two proteins were presented on the membrane. Cytoplasmic proteins can be considered for small molecule drug development while membrane or secreted proteins for vaccine development. On the other hand, the number of trans-membrane helices (Table 2) was set to be less or not more than 1. Proteins that were identified to have more than one trans-membrane helices were excluded due to the difficulties in cloning, expression and purification of proteins exhibiting multiple trans-membrane spanning regions.

**Table 2:** Identification of durggable targets characteristics and cellular localization.

S.No	UNIPORT ID	PROTEIN NAME	TMHMM No.	Molecular weight	Gene	LOCATION PSORTb	CELLO v 2.5	Query length
**Metabolic Pathway independent proteins**								
1	B0VT39	DNA gyrase subunit A	0	99.270	gyrA	Cytoplasm	Cytoplasm	904
2	B0VLK7	Bifunctional purine biosynthesis protein PurH	0	56.037	PurH	Cytoplasm	Cytoplasm	524
3	B0VUY5	Ferredoxin–NADP + reductase	0	29.361	Fpr	Cytoplasm	Cytoplasm	259
4	B0VTY4	Malonyl CoA-acyl carrier protein transacylase	0	34.494	fabD	Cytoplasm	Periplasmic, Cytoplasm	328
5	B0VSM6	Dihydropteroate synthase	0	30.848	folP	Cytoplasm	Cytoplasm	283
6	B0VTR5	Putative D-ala-D-ala-carboxypeptidase, penicillin-binding protein	0	48.899	ABSDF0993	Cytoplasmic membrane	Inner membrane, periplasmic	439
7	B0VTM9	Pyridoxine 5′-phosphate synthase	0	25.895	polxJ	Cytoplasm	Cytoplasm	241
8	B0VT18	Putative Oxidoreductase, short chain dehydrogenase/reductase family	0	28.797	ABSDF0838	Cytoplasm	Cytoplasm, outer membrane	260
9	B0VMS1	Putative UDP-glucose 6-dehydrogenase (Ugd) (Udg)	0	47.099	ABSDF0080	Cytoplasm	Cytoplasm	420
10	B0VNG2	Putative acetyl-coA synthetase/AMP-(Fatty) acid ligase	0	60.541	Absdf0123	Cytoplasm	Periplasmic, cytoplasmic	549
11	B0VTY3	3-oxoacyl-[acyl-carrier-protein] reductase	0	26.099	fabG	Unknown	cytoplasmic	244
**Metabolic pathway dependent proteins**								
1	B0VUZ6	Penicillin-binding protein 1B OS (Peptidoglycan biosynthesis)	0	88.189	mrcB	Unknown	Outer membrane	798
2	B0VMD0	Channel-tunnel spanning the outer membrane and periplasm segregation of daughter chromosomes (Bacterial secretion system, two component system, beta lactam resistance and CAMP resistance)	0	50.243	tolC	Outer membrane	Outer membrane	448

### Selection of antigenic membrane proteins

After the prioritization of target proteins, we found the four proteins (B0VUZ6, penicillin-binding protein 1B; B0VMD0, channel-tunnel spanning the outer membrane and periplasm segregation of daughter chromosomes; B0VTR5, putative D-ala-D-ala carboxypeptidase penicillin-binding protein; and B0VT18, putative oxidoreductase, short-chain dehydrogenase/reductase family) were present on the membrane hence considered for vaccine candidate molecules. VaxiJen web server showed that B0VUZ6 and B0VMD0 were identified as the most potent antigenic protein having a maximum total prediction score of 0.5541 and 0.5789 respectively (Supplementary Table ST-3). B0VMD0 is a protein involved in virulence while B0VUZ6 is an essential protein of *A. baumannii*. These two proteins with diverse role, and may be better situated for the development of vaccine candidates as they inhibit virulence as well as essential for the normal survival of *A. baumannii*. Hence, these two proteins are used for development of chimeric subunit vaccine against *A. baumannii*.

### Intra-species interaction of selected membrane proteins with other proteins

**Figure 3:**
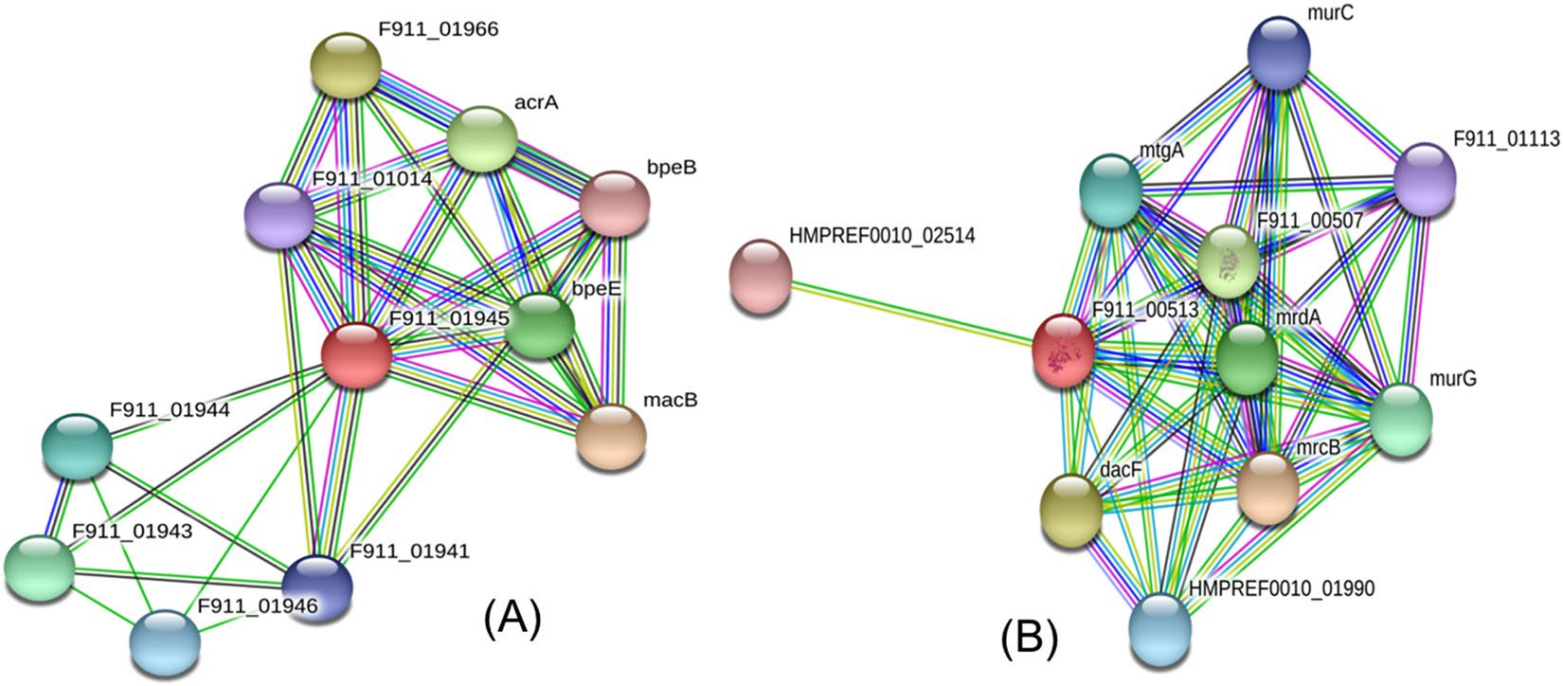
Protein-protein interaction diagram of (**A**) Channel-tunnel spanning the outer membrane and periplasm segregation of daughter chromosomes (**B**) penicillin binding protein predicted by STRING tool.

Penicillin binding protein 1A family protein (Fig. 3B) show interaction with the mrcB (murein polymerase), Dac F(serine-type D-Ala-D-Ala carboxypeptidase), F911_00507 (penicillin binding protein, transpeptidase domain protein), mrdA (penicillin binding protein 2), murG (UDP-diphospho-muramoylpentapeptide-beta-N-acetylgluco-saminyltransferase), mtgA(mono-functional biosynthetic peptidoglycan transglycosylase), HMPREF0010_01990 (serine type D-Ala-D-Ala carboxypeptidase), murC (UDP-N-acetylmuramate-alanine ligase), F911_01113 (uncharacterized proteins), and HMPREF0010_02514 (Type IV pilus assembly protein PilM). Out of these, murG, murC, and mtgA play role in cell wall formation and mrcB, plays a role not only in cell wall formation but also in the synthesis of cross-linked peptidoglycan from the lipid intermediates. Similarly, channel-tunnel spanning the outer membrane and periplasm segregation of daughter chromosomes protein (Fig. 3A) exhibited interactions with macB (macrolide export ATP binding/permease protein), F911_01966 (putative acriflavine resistance protein A), acrA (acriflavine resistance protein A), bpeE (multidrug efflux pump BpeE), F911_01943 (uncharacterized protein), F911_01944 (GH3 auxin-responsive promoter), F911_01946 (uncharacterized protein), F911_01941 (efflux pump membrane protein), F911_01014 (putative ATP synthase F0,A subunit) and bpeB (inner membrane multidrug efflux protein BpeB). These protein are very crucial in the survival of the *A. baumannii*. Therefore, targeting these two proteins (B0VUZ6 and B0VMD0) also influence the interacting proteins.

### Selection of potent T-cell epitopes in proteins shortlisted for chimeric vaccine design

On the basis of the high combinatorial score, best T-cell epitopes were predicted by NetCTL server using protein sequence of these two selected proteins. The software identified 17 epitopes in B0VMD0 proteins while 27 epitopes in B0VUZ6 (Supplementary Table ST-4). Peptides with a higher score represent higher processing capabilities. T-cell epitopes of both proteins were subjected to MHC-I binding prediction, using the IEDB server. The epitopes that elicited higher affinity (IC <200 nM) and percentile rank (≤0.2) were subjected to afterward analysis (Supplementary Table ST-5). These epitopes were further filtered on the basis of their interaction with class I MHC molecules. On the basis of MHC I molecules interaction, 10 out of 17 epitopes of B0VMD0 antigenic protein and 12 out of 27 epitopes of B0VUZ6 were selected for further analysis.

### Selection of Immunogenicity in selected T-cell epitopes

Besides the epitope predictions, the binding affinity between peptide/MHC complex and TCR was further analysed. A high immunogenicity score is deemed to have high ability to stimulate naive T cells and induce cellular immunity. The epitopes selected above (10 from B0VMD0 and 12 from B0VUZ6) using the above methods were subjected to IEDB immunogenicity prediction. The epitopes’ immunogenicity scores ranged from 0.24661 to −0.04637. In this analysis, 6 out of 10 epitopes of B0VMD0 protein and 8 out of 12 epitopes of B0VUZ6 contain the positive value score. These 14 (6 + 8) epitopes were selected for the further analysis (Supplementary Table ST-5).

### Toxicity, conservancy and antigenicity prediction of selected 14 epitopes

To confirm the specific immune responses induced by epitopes, ToxinPred was used to confirm epitopes non-toxicity. Epitopes toxicity analysis score (Table 3) confirmed that the selected 14 epitopes were non-toxic. Conservancy status of selected epitopes of B0VMD0 and B0VUZ6 were determined using IEDB conservancy analysis. Epitope which showed more than 50% conservancy status were selected for further analysis. The result showed that three epitopes of B0VMD0 like DVSEANAQY (70%), SSSFALDLV(60%) and HVLNVAEAY(60%) are conserved in 10 other genotype sequences. Similarly, in the protein B0VUZ6, all the 8 epitopes showed conservancy score more than 50% such as MALLIELHY (96.49%), ISTEDRNFY (96.49%), LSAIESGRY (90.35%), STFTNNLRK (85.56%), LLDRYGLNV (92.98%), SSGTGRAAY (85.96), GVESTIPAY (70.18) and YVHTRGFDY (60.53%) and found to be conserved in 114 other genotype sequences. Potent epitope antigenicity of selected 11 epitopes (3 of B0VMD0 and 8 of B0VUZ6) were analysed using VaxiJen web server. Result showed that, a total of seven epitopes (2 of B0VMD0 such as DVSEANAQY, SSSFALDLV, and 5 of B0VUZ6 such as MALLIELHY, LLDRYGLNV, SSGTGRAAY, ISTEDRNFY and YVHTRGFDY) contained the potent antigenicity (Table 3). These 7 epitopes were selected for further study.

**Table 3:** Predicted MHC class I epitopes Toxicity, Antigenicity and conservancy analysis.

S.No	Protein name	Peptide	Toxicity (SVM score)	Antigenicity	Conservancy (%)
1	B0VMD0 Channel-tunnel spanning the outer membrane and periplasm segregation of daughter chromosomes	DVSEANAQY	Non toxic (−0.70)	0.9791	70.00%
		SSSFALDLV	Non toxic (−0.59)	0.5339	60.00%
		FALDLVETY	Non toxic (−1.04)	0.0302	40.00%
		HVLNVAEAY	Non toxic (−1.31)	−0.2143	60.00%
		RQQALTAAY	Non toxic (−1.11)	0.3587	30.00%
		QLSEYIGPY	Non toxic (−0.60)	−0.7155	20.00%
2	B0VUZ 6Penicillin-binding protein 1B OS	MALLIELHY	Non toxic (−1.28)	1.3841	96.49%
		LSAIESGRY	Non toxic (−0.78)	0.0863	90.35%
		STFTNNLRK	Non toxic (−1.15)	0.1311	85.96%
		LLDRYGLNV	Non toxic (−1.23)	1.4214	92.98%
		SSGTGRAAY	Non toxic (−0.58)	1.7890	85.96%
		GVESTIPAY	Non toxic (−1.20)	0.4407	70.18%
		ISTEDRNFY	Non toxic (−1.51)	0.8503	96.49%
		YVHTRGFDY	Non toxic (−0.61)	1.7617	60.53%

### Selection of MHC-II epitopes in proteins shortlisted for chimeric vaccine design and their conservancy analysis

In addition to MHC-I epitope predication, both the selected proteins (B0VMD0 and B0VUZ6) were further subjected to MHC-II binding prediction, using IEDB server. The epitopes that elicited higher affinity (IC <200 nM) and low percentile rank were subjected to afterward analysis (Table 4). Conservancy analysis showed that two epitopes of B0VMD0 showed the conservancy more than 50% such as LEQLNMMNAKLKEGL (60%) and MVDVLLAQRNAFSAK (70%) in 10 other genotype sequences. Similarly, six epitopes of B0VUZ6 showed the conservancy more than 50% which include FERGIGFFALIFSIL (84.21%), ALSIYLIRLDNIIRE (92.11%), GRAAYNSLSPALKLA (64.04%), LSTFTNNLRKFGVES (85.96%), FTGFNRALDAKRQVG (78.01%), STEDRNFYHHHGISI (85.09%). These 8 epitopes (2 of B0VMD0 and 6 of B0VUZ6) were selected for further analysis.

**Table 4:** Predicted of MHC class II epitopes by IEDB server and conservancy analysis.

S.N	B0VMD0 epitopes	Start	HLA ALLELES (Percentile Rank)	Conservancy	B0VUZ6 epitopes	Start	HLA ALLELES (Percentile Rank)	Conservancy
1	AVLRSDFIFQKPYPA	229	HLA-DRB3*01:01 (0.02)	30.00%	FERGIGFFALIFSIL	3	HLA-DPA1*01:03/DPB1*02:01(0.01)	84.21%
2	LEQLNMMNAKLKEGL	169	HLA-DRB5*01:01 (0.02)	60.00%	ALSIYLIRLDNIIRE	24	HLA-DRB3*01:01 (0.02)	92.11%
3	VLRQQALTAAYLQEE	151	HLA-DQA1*04:01/DQB1*04:02(0.04)	30.00%	GRAAYNSLSPALKLA	624	HLA-DRB5*01:01 (0.02)	64.04%
4	SARQPLFRMDAWEGY	105	HLA-DRB3*01:01 (0.14)	30.00%	AFKASVERLANSNPA	397	HLA-DRB1*09:01 (0.06)	37.72%
5	MVDVLLAQRNAFSAK	395	HLA-DRB1*03:01 (0.19)	70.00%	LSTFTNNLRKFGVES	528	HLA-DRB1*11:01 (0.11)	85.96%
6	MKIKLMLVAGLWSFT	1	HLA-DRB4*01:01 (0.18)	30.00%	FTGFNRALDAKRQVG	440	HLA-DRB5*01:01 (0.13)	78.07%
7	—	—	—	—	STEDRNFYHHHGISI	175	HLA-DRB1*07:01 (0.15)	85.09%
8	—	—	—	—	AQFYFGLPLRELNVA	274	HLA-DPA1*02:01/DPB1*14:01(0.16)	0.88%

### MHC restriction and cluster analysis of selected epitopes

**Figure 4:**
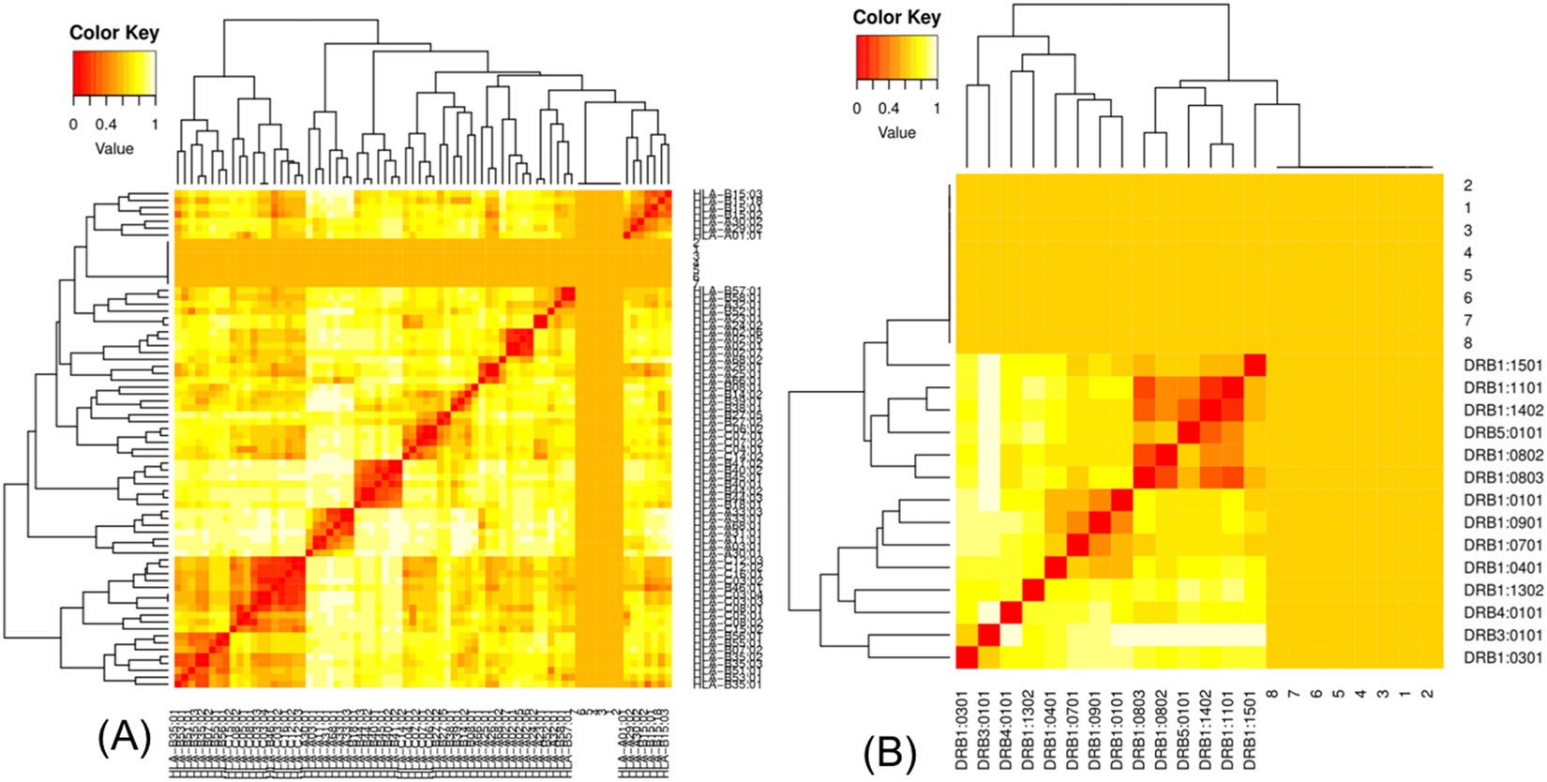
Cluster analysis of the HLA alleles for both MHC molecules through heat map representation. (**A**) Representing the cluster of the MHC-I. (**B**) Representing the cluster of MHC-II molecules. Epitopes are clustered on the basis of interaction with HLA and shown as red colour indicating strong interaction with appropriate annotation. Yellow zone indicates the weaker interaction. Here, all the available alleles are shown.

Further, MHC class I and MHC class II restricted allele were further analysed on the basis of the IC_50_value. All the predicted epitopes were assessed for the MHC interaction analysis independently for 7 MHC-1 epitope and 8 MHC-II epitopes. Furthermore, the interacted alleles were reassessed by cluster analysis and results are shown as a heat map of MHC-1 (Fig. 4A) and MHC-II (4B), and dynamic tree (Supplementary Figure SF-2). Epitopes shown in Fig. 4 are clustered on the basis of interaction with different HLA alleles and red colour indicating strong interactions while yellow colour shows weaker interactions with appropriate annotation^63^.

### Selection of B-cell epitopes in proteins shortlisted for chimeric vaccine design

**Figure 5:**
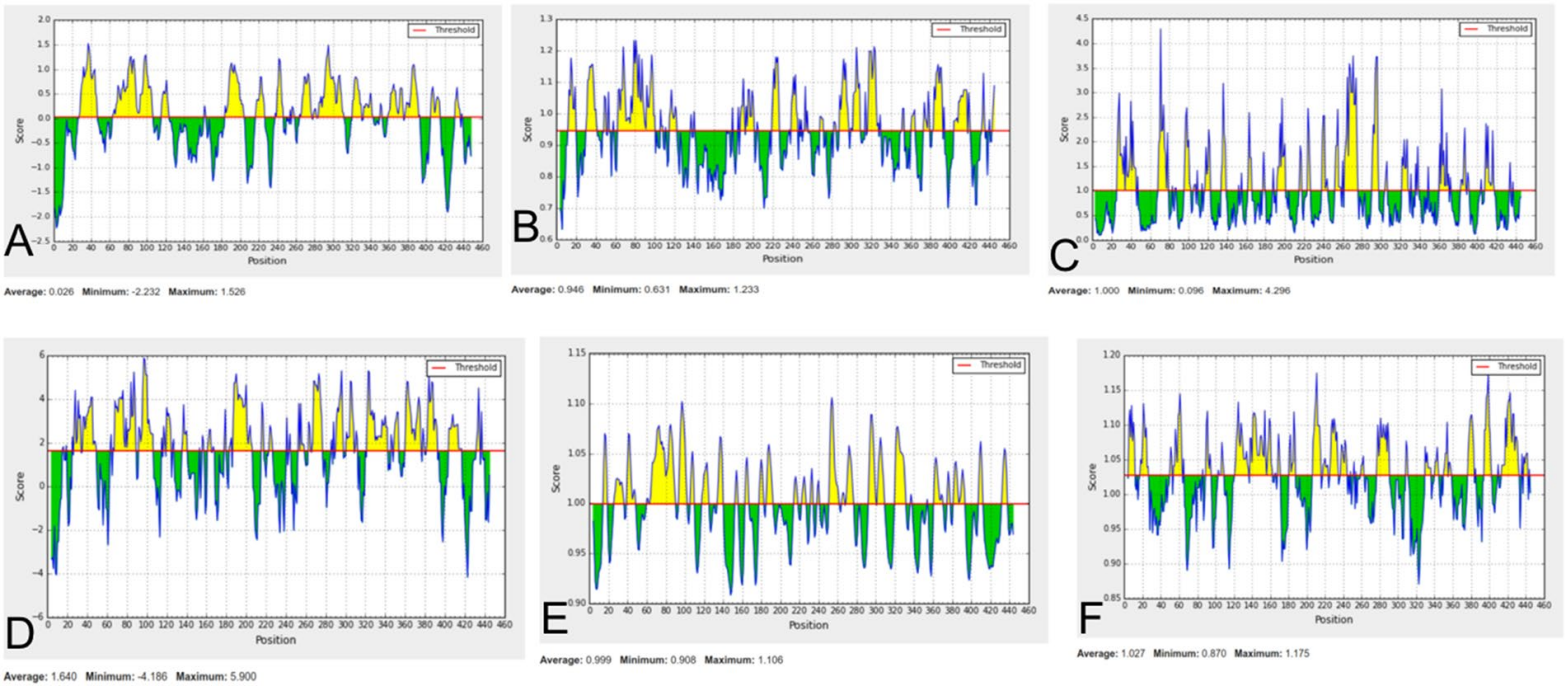
Protein ID B0VMD0 B cell epitope (**A**) Bepipred Linear Epitope, (**B**) Chou & Fasman Beta-Turn Prediction, (**C**) Emini Surface Accessibility Prediction, (**D**) Karplus & Schulz Flexibility Prediction, (**E**) Kolaskar & Tongaonkar Antigenicity, (**F**) Parker Hydrophilicity Prediction.

**Figure 6:**
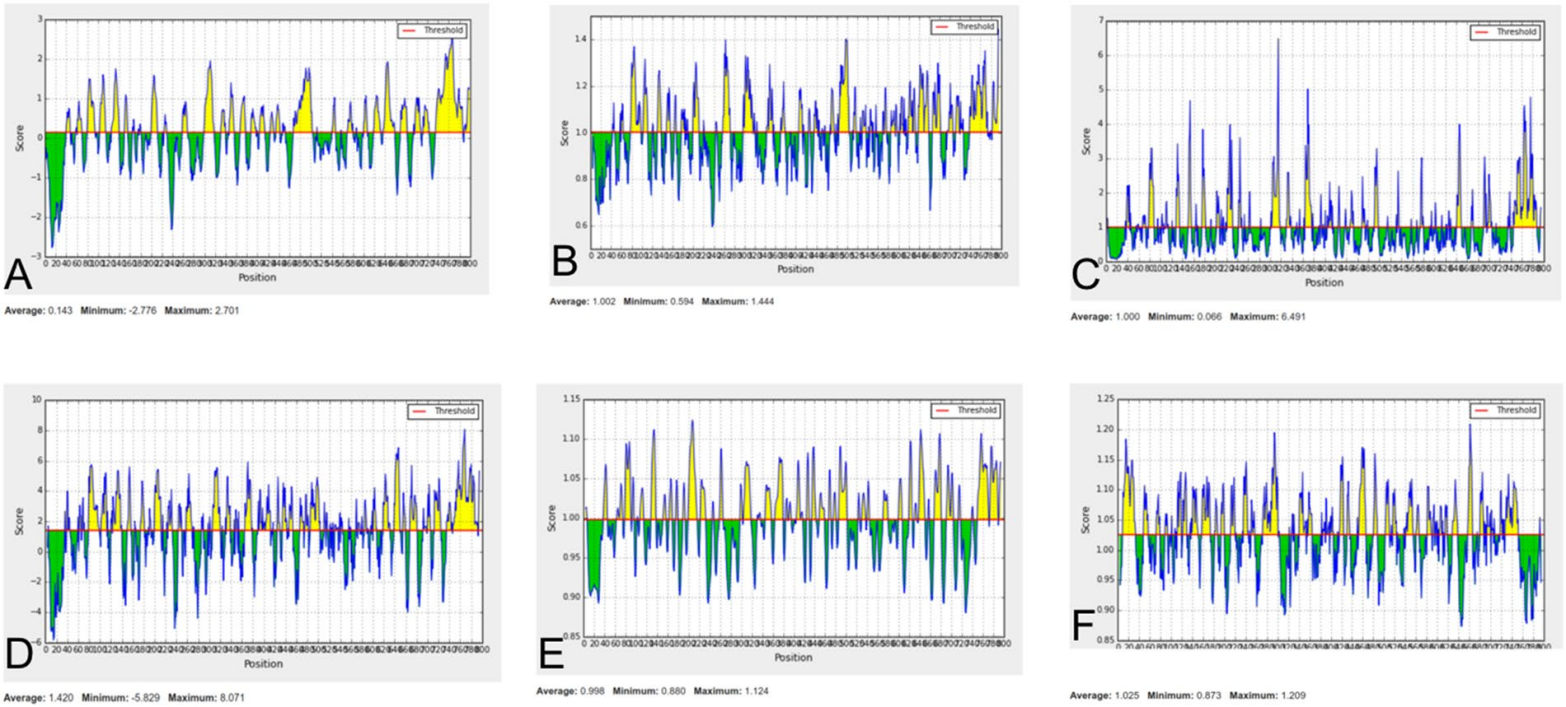
Protein ID B0VUZ6 B cell epitope (**A**) Bepipred Linear Epitope, (**B**) Chou & Fasman Beta-Turn Prediction, (**C**) Emini Surface Accessibility Prediction, (**D**) Karplus & Schulz Flexibility Prediction, (**E**) Kolaskar & Tongaonkar Antigenicity, (**F**) Parker Hydrophilicity Prediction.

In addition to MHC-I and MHC-II epitopes (cellular immunity), these two proteins were also used to predict the B-cell epitope (humoral immunity). Humoral immunity is also required for bacterial elimination. To predict B-cell epitopes, firstly location of linear B-cell epitopes was identified using BCPREDS, FBCpred, and IEDB (Supplementary Tables ST6 and ST7). Resulted epitopes were further shortlisted on the basis of BepiPred linear epitope prediction, Karplus-Schulz flexibility prediction, Chou-Fasman beta-turn prediction, Kolaskar Tongaonkar antigenicity, Emini surface accessibility prediction, and Parker hydrophilicity prediction. The result of predicated B-cell epitopes of B0VMD0 and B0VUZ6 are shown in Figs 5 and 6 respectively. Interaction of shortlisted 10 B-cell epitopes (4 of B0VMD0 and 6 of B0VUZ6) with the HLA-II allele’s protein was performed using IEDB and result is shown in Supplementary Table ST-8. In the result, highest binding HLA alleles were selected on the basis of percentile rank (<0.2) and IC_50_ value (<200 nM).

### Comparison of all the predicted epitopes to select the final epitopes

For the construction of final chimeric subunit vaccine sequence, predicted B-cell epitopes were treated as a template for MHC-I, and MHC-II epitopes and those epitopes whose sequences were found to be overlapping in B-cell epitopes were shortlisted and selected for final vaccine constructs (Supplementary Tables 9 and 10).

### Construction of final vaccine with different adjuvants, linkers, and PADRE sequence

An adjuvant is an ingredient of a vaccine that helps create a stronger immune response in the patient’s body. Different adjuvants were chosen in the vaccine constructs to analyze the effects of the adjuvants on the allergenicity and antigenicity of the constructs. Along with adjuvants sequence, PADRE sequence was also added to overcome the problems caused by polymorphism of HLA-DR molecules in the worldwide population. It showed the 100-fold more potency than the universal T helper epitopes^64^, and it was explained that PADRE containing vaccine construct exhibited better CTL responses and protection than the vaccine without the T helper epitope^64^. A total of eight vaccine constructs were prepared and further analyzed. All eight vaccine constructs were added with the respective adjuvants with the help of EAAAK linker. MHC-I, MHC-II, B-epitopes and PADRE sequence were joined together by HEYGAEALERAG and GGGS linkers. All the epitopes were joined together with these two linkers because they are reported not to alter the conformation of designed vaccine construct^55^. Details of the vaccine constructs have been mentioned in Table 5.

**Table 5:** Allergenicity prediction of all vaccine construct using Algpred server.

S. No.	Vaccine constructs	Epitope sequence position with adjuvant	Complete sequence of vaccine construct	Allergenicity (Algpred) (Threshold-0.4)
1.	V1	BOVMD0 (62–106, 359–419), and BOVUZ6 (688–787, 79–139, 366–382) epitope with HBHA adjuvant and PADRE sequence	**EAAAK**MAENPNIDDLPAPLLAALGAADLALATVNDLIANLRERAEETRAETRTRVEERRARLTKFQEDLPEQFIELRDKFTTEELRKAAEGYLEAATNRYNELVERGEAALQRLRSQTAFEDASARAEGYVDQAVELTQEALGTVASQTRAVGERAAKLVGIEL**EAAAK***AKFVAAWTLKAAA***GGGS**VTLSGNITRNRQTVKRSNFPGVDQEGLSDALVSNTSTTKQATLSA**GGGS**QVDTDRAKLEARAAMDSSALVSQASKASYNEGLKSMVDVLLAQRNAFSAKQDYLNAQYDYL**GGGS***AKFVAAWTLKAAA***GGGS**TPVNLRQPDSVQWQWIDHASGDLSAQACDGAMYIPMLAHTVPHRATPCGAPYYQVDPTYTPQSDNTIPEPEDDNTDSYIRESENQMEQDLSNNTRIISSG**HEYGAEALERAG**KTSSNYDKSGTYVAQGSNMYVHTRGFDYGDSVEPEQVLELSFANDQVVEVRSTKPSSTGVA**HEYGAEALERAG**RTEYQESDLTNQGLRI**HEYGAEALERAG***AKFVAAWTLKAAA***GGGS**	−0.6597
2	V2	BOVMD0 (62–106, 359–419), and BOVUZ6 (688–787, 79–139, 366–382) epitope with HBHA conserved adjuvant and PADRE sequence	**EAAAK**MAENSNIDDIKAPLLAALGAADLALATVNELITNLRERAEETRRSRVEESRARLTKLQEDLPEQLTELREKFTAEELRKAAEGYLEAATSELVERGEAALERLRSQQSFEEVSARAEGYVDQAVELTQEALGTVASQVEGRAAKLVGIEL**EAAAK***AKFVAAWTLKAAA***GGGS**VTLSGNITRNRQTVKRSNFPGVDQEGLSDALVSNTSTTKQATLSA**GGGS**QVDTDRAKLEARAAMDSSALVSQASKASYNEGLKSMVDVLLAQRNAFSAKQDYLNAQYDYL**GGGS***AKFVAAWTLKAAA***GGGS**TPVNLRQPDSVQWQWIDHASGDLSAQACDGAMYIPMLAHTVPHRATPCGAPYYQVDPTYTPQSDNTIPEPEDDNTDSYIRESENQMEQDLSNNTRIISSG**HEYGAEALERAG**KTSSNYDKSGTYVAQGSNMYVHTRGFDYGDSVEPEQVLELSFANDQVVEVRSTKPSSTGVA**HEYGAEALERAG**RTEYQESDLTNQGLRI**HEYGAEALERAG***AKFVAAWTLKAAA***GGGS**	−0.66619
3	V3	BOVMD0 (62–106, 359–419), and BOVUZ6 (688–787, 79–139, 366–382) epitope with beta defensin adjuvant and PADRE sequence	**EAAAK**GIINTLQKYYCRVRGGRCAVLSCLPKEEQIGKCSTRGRKCCRRKK**EAAAK**AKFVAAWTLKAAA**GGGS**VTLSGNITRNRQTVKRSNFPGVDQEGLSDALVSNTSTTKQATLSA**GGGS**QVDTDRAKLEARAAMDSSALVSQASKASYNEGLKSMVDVLLAQRNAFSAKQDYLNAQYDYL**GGGS***AKFVAAWTLKAAA***GGGS**TPVNLRQPDSVQWQWIDHASGDLSAQACDGAMYIPMLAHTVPHRATPCGAPYYQVDPTYTPQSDNTIPEPEDDNTDSYIRESENQMEQDLSNNTRIISSG**HEYGAEALERAG**KTSSNYDKSGTYVAQGSNMYVHTRGFDYGDSVEPEQVLELSFADQVVEVRSTKPSSTGVA**HEYGAEALERAG**RTEYQESDLTNQGLRI**HEYGAEALERAG***AKFVAAWTLKAAA***GGGS**	−0.5014
4	V4	BOVMD0 (162–204, 231–336), and B0VUZ6 (174–248, 636–659) with beta defensin adjuvant and PADRE sequence	**EAAAK**GIINTLQKYYCRVRGGRCAVLSCLPKEEQIGKCSTRGRKCCRRKK**EAAAK***AKFVAAWTLKAAA***GGGS**LQEEKALLEQLNMMNAKLKEGLVARSDVSEANAQYQNARANRI**GGGS**LRSDFIFQKPYPAQLDEWLGLTQQQNLKIQQARLQKRYAEDQRRVEKEKAALYPQIDAVASYGYTKQTPETLISTDGKFDQGVEMNWNLFNGGRTRTSIKKASVELN**HEYGAEALERAG**ISTEDRNFYHHHGISIRGTARALVSNVTGGRRQGGSSTLTQQLVKNFYLTPERTLKRKVNEALMALLIELHYSKDE**HEYGAEALERAG**KLAGKSGTTNDTRDSWFAGYSGN**HEYGAEALERAG***AKFVAAWTLKAAA***GGGS**	−0.4578
5	V5	BOVMD0 (162–204, 231–336), and B0VUZ6 (174–248, 636–659) with HBHA adjuvant and PADRE sequence	**EAAAK**MAENPNIDDLPAPLLAALGAADLALATVNDLIANLRERAEETRAETRTRVEERRARLTKFQEDLPEQFIELRDKFTTEELRKAAEGYLEAATNRYNELVERGEAALQRLRSQTAFEDASARAEGYVDQAVELTQEALGTVASQTRAVGERAAKLVGIEL**EAAAK***AKFVAAWTLKAAA***GGGS**LQEEKALLEQLNMMNAKLKEGLVARSDVSEANAQYQNARANRI**GGGS**LRSDFIFQKPYPAQLDEWLGLTQQQNLKIQQARLQKRYAEDQRRVEKEKAALYPQIDAVASYGYTKQTPETLISTDGKFDQGVEMNWNLFNGGRTRTSIKKASVELN**HEYGAEALERAG**ISTEDRNFYHHHGISIRGTARALVSNVTGGRRQGGSSTLTQQLVKNFYLTPERTLKRKVNEALMALLIELHYSKDE**HEYGAEALERAG**KLAGKSGTTNDTRDSWFAGYSGN**HEYGAEALERAG***AKFVAAWTLKAAA***GGGS**	−0.3
6	V6	BOVMD0 (62–106, 359–419), and BOVUZ6 (688–787, 79–139, 366–382) epitope with L7/L12 Ribosomal protein adjuvant and PADRE sequence	**EAAAK**MAKLSTDELLDAFKEMTLLELSDFVKKFEETFEVTAAAPVAVAAAGAAPAGAAVEAAEEQSEFDVILEAAGDKKIGVIKVVREIVSGLGLKEAKDLVDGAPKPLLEKVAKEAADEAKAKLEAAGATVTVK**EAAAK***AKFVAAWTLKAAA***GGGS**VTLSGNITRNRQTVKRSNFPGVDQEGLSDALVSNTSTTKQATLSA**GGGS**QVDTDRAKLEARAAMDSSALVSQASKASYNEGLKSMVDVLLAQRNAFSAKQDYLNAQYDYL**GGGS***AKFVAAWTLKAAA***GGGS**TPVNLRQPDSVQWQWIDHASGDLSAQACDGAMYIPMLAHTVPHRATPCGAPYYQVDPTYTPQSDNTIPEPEDDNTDSYIRESENQMEQDLSNNTRIISSG**HEYGAEALERAG**KTSSNYDKSGTYVAQGSNMYVHTRGFDYGDSVEPEQVLELSFANDQVVEVRSTKPSSTGVA**HEYGAEALERAG**RTEYQESDLTNQGLRI**HEYGAEALERAG***AKFVAAWTLKAAA***GGGS**	0.0460
7	V7	BOVMD0 (162–204, 231–336), and B0VUZ6 (174–248, 636–659) with L7/L12 Ribosomal protein adjuvant and PADRE sequence	**EAAAK**MAKLSTDELLDAFKEMTLLELSDFVKKFEETFEVTAAAPVAVAAAGAAPAGAAVEAAEEQSEFDVILEAAGDKKIGVIKVVREIVSGLGLKEAKDLVDGAPKPLLEKVAKEAADEAKAKLEAAGATVTVK**EAAAK***AKFVAAWTLKAAA***GGGS**LQEEKALLEQLNMMNAKLKEGLVARSDVSEANAQYQNARANRI**GGGS**LRSDFIFQKPYPAQLDEWLGLTQQQNLKIQQARLQKRYAEDQRRVEKEKAALYPQIDAVASYGYTKQTPETLISTDGKFDQGVEMNWNLFNGGRTRTSIKKASVELN**HEYGAEALERAG**ISTEDRNFYHHHGISIRGTARALVSNVTGGRRQGGSSTLTQQLVKNFYLTPERTLKRKVNEALMALLIELHYSKDE**HEYGAEALERAG**KLAGKSGTTNDTRDSWFAGYSGN**HEYGAEALERAG***AKFVAAWTLKAAA***GGGS**	0.113
8	V8	BOVMD0 (162–204, 231–336), and B0VUZ6 (174–248, 636–659) with HBHA adjuvant and PADRE sequence	**EAAAK**MAENPNIDDLPAPLLAALGAADLALATVNDLIANLRERAEETRAETRTRVEERRARLTKFQEDLPEQFIELRDKFTTEELRKAAEGYLEAATNRYNELVERGEAALQRLRSQTAFEDASARAEGYVDQAVELTQEALGTVASQTRAVGERAAKLVGIEL**EAAAK***AKFVAAWTLKAAA***GGGS**LQEEKALLEQLNMMNAKLKEGLVARSDVSEANAQYQNARANRI**GGGS**LRSDFIFQKPYPAQLDEWLGLTQQQNLKIQQARLQKRYAEDQRRVEKEKAALYPQIDAVASYGYTKQTPETLISTDGKFDQGVEMNWNLFNGGRTRTSIKKASVELN**HEYGAEALERAG**ISTEDRNFYHHHGISIRGTARALVSNVTGGRRQGGSSTLTQQLVKNFYLTPERTLKRKVNEALMALLIELHYSKDE**HEYGAEALERAG**KLAGKSGTTNDTRDSWFAGYSGN**HEYGAEALERAG***AKFVAAWTLKAAA***GGGS**	−0.32975

### Allergenicity, antigenicity and solubility prediction of different vaccine constructs

AlgPred server was used to predict the non-allergic behaviour of vaccine constructs. Allergenicity value of all eight vaccine constructs were shown (Table 5). Predicted vaccine constructs score indicates that four constructs (first vaccine construct with HBHA adjuvant (V1), with HBHA conserved sequence (V2), with beta-defensin (V3) as well as second vaccine construct with beta-defensin (V4) were found to be non-allergic in nature. Antigenicity of shortlisted four non-allergenic vaccine constructs (V1, V2, V3, V4) were further predicted using ANTIGENpro and followed by VaxiJen 2.0. (Supplementary Table ST-11). The predicted antigenicity values were more than 0.90 in ANTIGENpro and 0.75 in VaxiJen 2.0 that shows a good antigenic nature of these four vaccine constructs. SOLpro server predicts the protein solubility and corresponding probability should be ≥0.5. All four vaccine constructs showed the solubility more than 0.7 (Supplementary Table 11), which exhibited that vaccine construct will be highly soluble during its heterologous expression in the *E. coli*.

### Physicochemical analysis of vaccine constructs

Various physicochemical properties such as molecular weight, number of amino acid, PI value, aliphatic index, hydropathicity index and instability index, of the all four constructs were calculated from ProtParam server (Supplementary Table 12). The molecular weights of constructs were found between 41–59 kDa that shows its good antigenic properties. GRAVY (a hydropathic index) was found to be less than −0.5 (a negative value), which indicates hydrophilic nature of constructs. Aliphatic and stability value also showed that all the four vaccine constructs have good characteristics to initiate an immunogenic reaction.

### Structure prediction of selected four vaccine constructs

**Figure 7:**
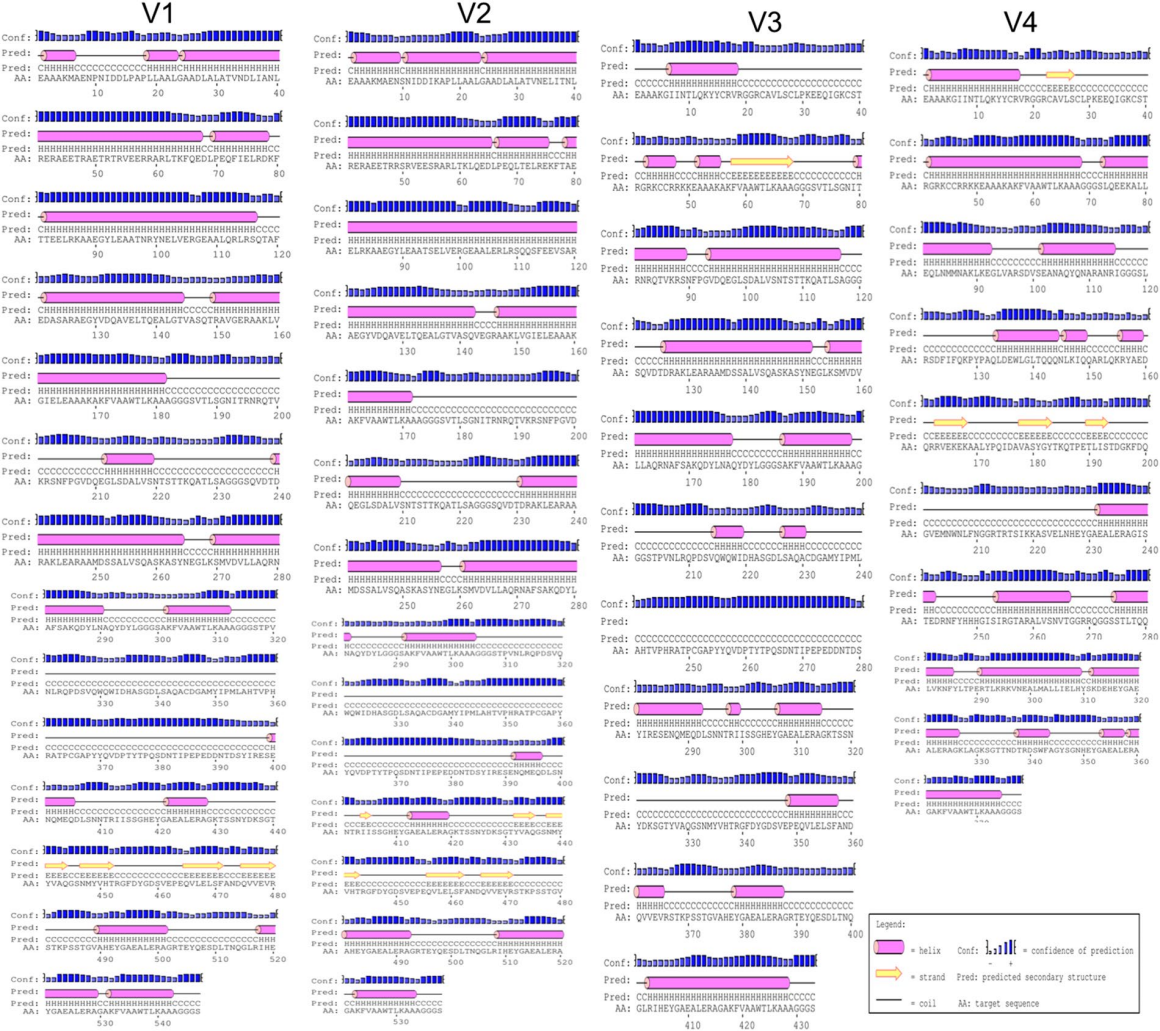
Secondary structure prediction of vaccine constructs (V1 to V4) using PESIPRED server.

**Figure 8:**
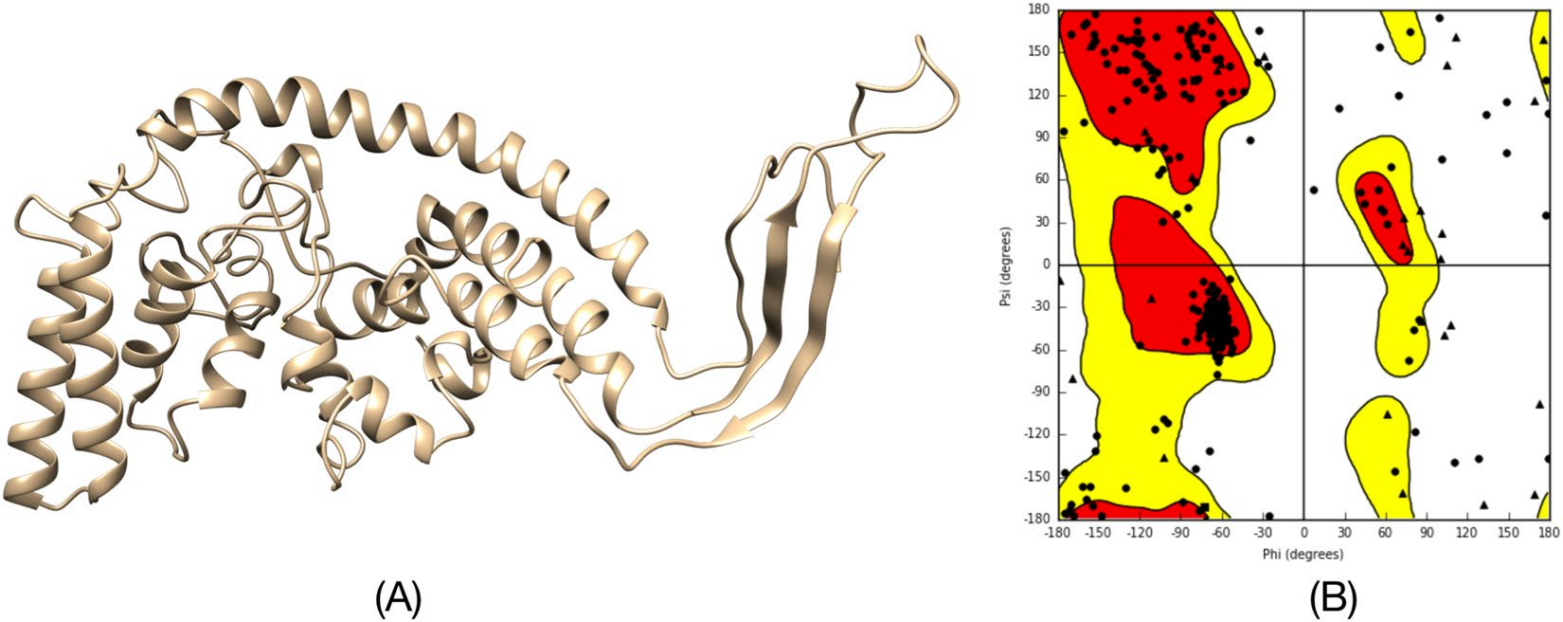
Tertiary Structure prediction and validation of vaccine construct V4. (**A**) Tertiary structure of model construct V4. (**B**) Ramachandran plot of the modelled V4 showing 92.0 residues in the allowed region.

Secondary structures of final four vaccine constructs were identified using PSIPRED. The predicted structure of all four vaccine constructs have shown to have 55–60% alpha helix, 10–15% extended strand, 7–12% beta turn, and 20–30% coil structure (Fig. 7). The models of all four vaccine constructs were generated and the Ramachandran plot was used to validate these models. Modeled tertiary structure of vaccine 4 and Ramachandran plot have been shown in Fig. 8.

### Molecular docking

#### Docking of vaccine constructs (V1 to V4) with HLA allele’s protein

Linear T-cell epitopes binds to HLA molecules and activate the adaptive immunity against pathogen. The immune response elicited against the epitopes may be genetically restricted or epitopes may be recognized by one individual but not by other^65^. Hence, a vaccine construct against *A. baumannii* should have a potential to induce immune response against the number of epitopes, which can be recognized in the context of different HLA allele’s proteins. Promiscuous vaccine can bind to different HLA allelic proportion of human populations. We have docked all four vaccine constructs with the six different HLA allele’s protein. Vaccine construct 4 (V4) have the lowest global binding energy value with different HLA alleles i.e. 2FSE (HLA-DR B1*01:01); −45.02, 2SEB (HLA-DR B1*04:01); −32.17, 1H1S (HLA-DR B5*01:01); −12.92, 1A6A (HLA-DR B1*03:01); −0.90, 3C5J (HLA-DR B3*02:02); −11.22, and 2Q6W (HLA-DR B3*01:01) −8.74 as shown in Table 6. On the basis of the different criteria, we have analysed all four different constructs and finalized the one suitable, and best vaccine construct i.e. V4 that can control *A. baumannii* infection.

**Table 6:** Docking score of different vaccine construct (V1 to V4) with the different HLA alleles.

Vaccine constructs	HLA alleles PDB ID’s^#^	SCORE	AREA	HYDROGEN BOND energy	GLOBEL ENERGY	ACE
V1	1A6A	18932	2545	−0.85	−11.67	11.75
	3C5J	18580	2590	−2.34	−13.31	3.00
	1H15	20000	3198	−0.97	4.32	1.34
	2FSE	21168	3000	−3.68	−11.45	11.58
	2Q6W	19340	2493	−1.98	−4.38	12.14
	2SEB	18614	2527	−5.55	−15.29	7.00
V2	1A6A	18714	2898	−4.37	−10.14	6.20
	3C5J	18582	2537	−3.12	−17.74	5.00
	1H15	18124	2814	−5.71	−29.89	2.77
	2FSE	19250	2914	−2.57	−9.80	3.75
	2Q6W	18072	2524	−4.94	−38.66	2.09
	2SEB	19168	3156	−3.93	−11.03	16.64
V3	1A6A	19264	2997	−3.26	−17.76	4.43
	3C5J	18552	2881	−2.24	−19.96	10.68
	1H15	19780	2391	−6.17	−14.91	9.40
	2FSE	22094	3178	−7.31	−14.80	18.68
	2Q6W	19988	4105	−6.62	−11.49	4.92
	2SEB	19030	2221	−1.80	−32.26	−9.60
V4	1A6A	18320	2672	−4.47	−0.90	13.41
	3C5J	17148	2335	−5.07	−11.22	8.26
	1H15	20660	3533	−1.67	−12.92	6.23
	2FSE	17310	2476	−7.78	−45.02	−0.52
	2Q6W	17460	2205	−4.25	−8.74	9.56
	2SEB	19030	2358	−4.84	−32.17	5.52

^#^1A6A(HLA-DR B1*03:01), 3C5J(HLA-DR B3*02:02), 1H15(HLA-DR B5*01:01), 2FSE(HLA-DR B1*01:01), 2Q6W(HLA-DR B3*01:01), 2SEB(HLA-DR B1*04:01).

#### Docking, and Molecular Dynamics Simulation of vaccine construct (V4) and TLR 4

**Figure 9:**
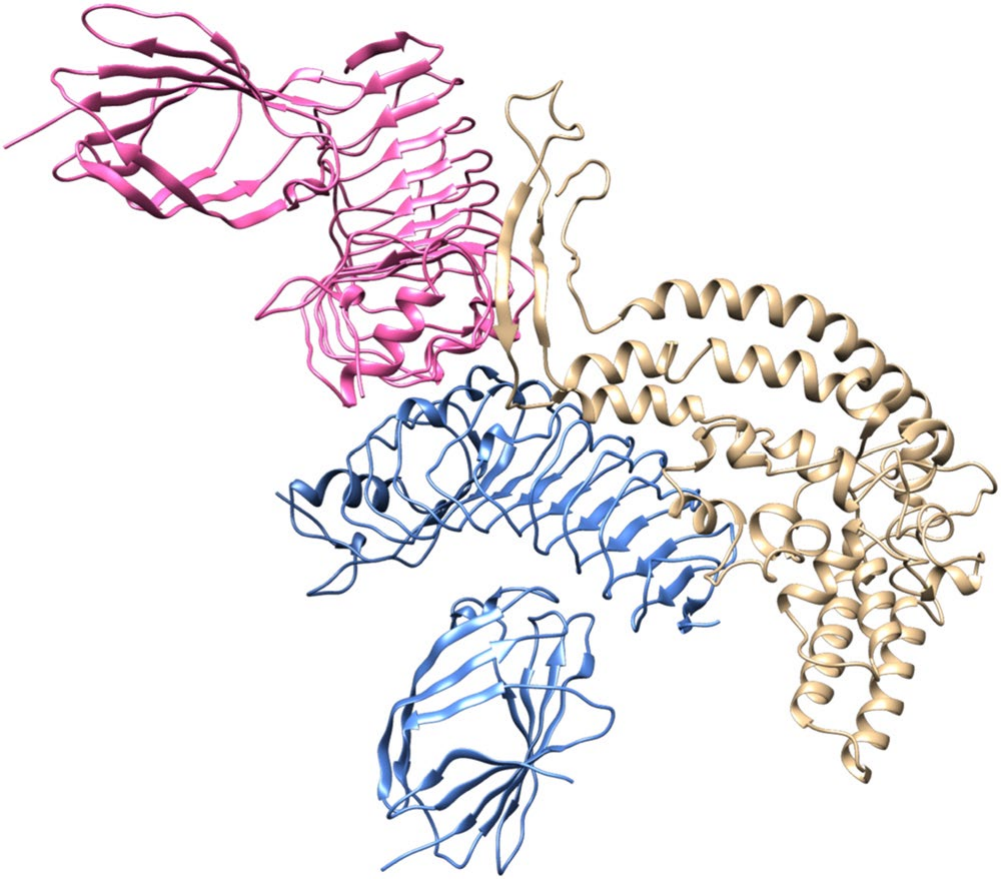
Docked complex of vaccine construct V4 with human TLR4-MD2 complex. The vaccine construct docked within the TLR-4 receptor.

**Figure 10:**
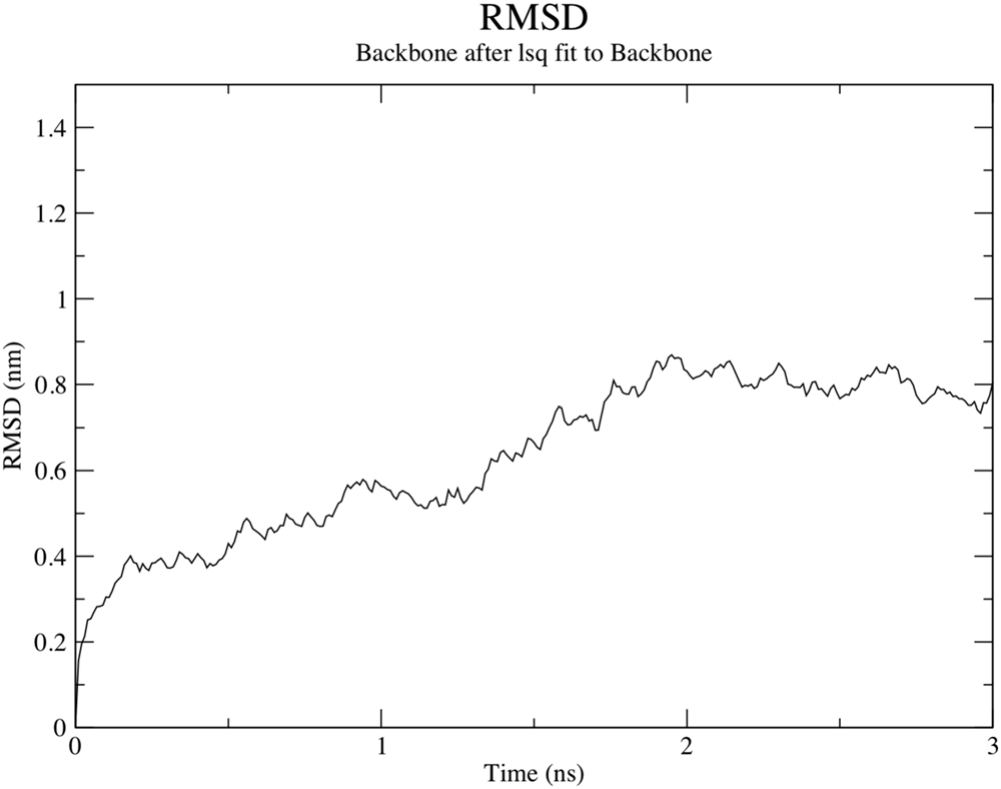
Molecular dynamics simulation of V4- TLR4-MD2 complex. The result shows the RMSD obtained for the complex which showed that complex is stable after 2 ns at 0.8 nm.

TLR 4 is agonist to beta-defensin. The interaction between the TLR4 and adjuvant enhance the immune response. Therefore, we have performed docking between vaccine construct (V4) with TLR 4. The result showed that docking score of 17970 (−31.11 binding energy) that showed good interaction between vaccine construct V4 and TLR-4/MD2 complex (Fig. 9). The molecular dynamics simulation confirms that V4-TLR-4/MD2 complex get stabilised after 2 ns and remain stable (Fig. 10).

### *In-silico* cloning of chimeric vaccine construct (V4) for its heterologous expression

**Figure 11:**
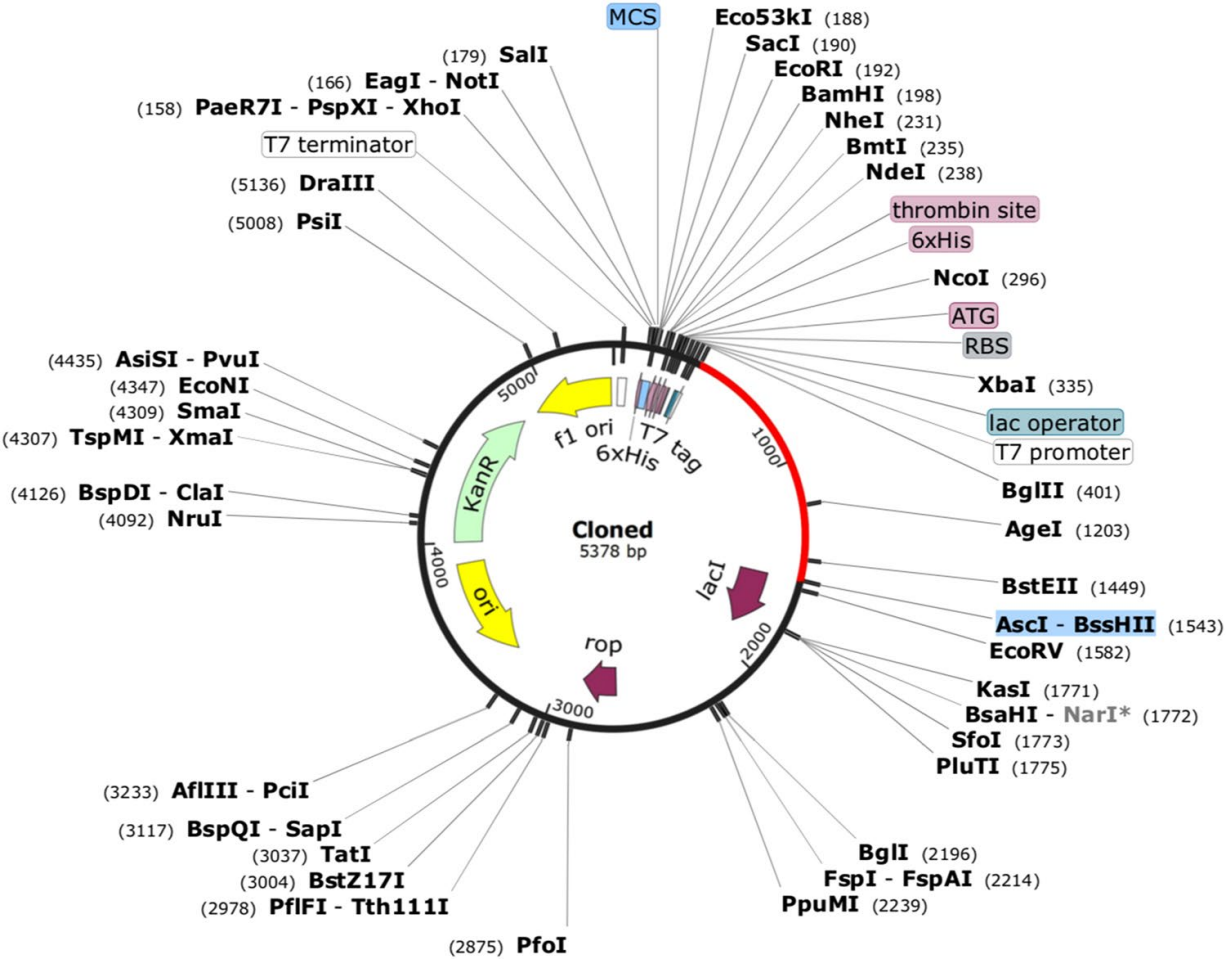
*In-silico* restriction cloning of gene sequence of final vaccine construct V4 into pET28a expression vector showing V4 sequence red colour surrounded between BglII (401) and AscI (1543).

Reverse translation and codon optimization were performed from Codon Usage Wrangler, in order to evaluate the cloning and expression of multi-epitope vaccine within the expression vector. Reverse translation generates cDNA sequence, which was further analyzed by codon optimization analysis. Codon optimization evaluates the sequence on the basis of GC content of the cDNA sequence. The optimum range was found to be between 30–70% for all the four constructs. This is followed by CAI calculation. The high value of CAI indicates high expression of the gene. Our constructs have the CAI value in the range of 0.95–1.0 (Supplementary Table ST-11). Final vaccine construct (V4) was cloned into pET28a vector for its heterologous cloning and expression in *E. coli* (Fig. 11).

## Discussion

*Acinetobacter baumannii* infection has emerged as a severe problem, reasons for a large number of deaths in worldwide. Despite this, there is no permanent cure and prevention for the multi drug resistant *A. baumannii*. Some approaches have been tried which includes the screening of herbal compounds^66–68^, nanomaterial based approach^69,70^ and *in-silico* approach^71,72^ to find an alternative to the current drug used against *A. baumannii*. Similarly, subtractive genomics of different strains of *A. baumannii* has also been tried to identify the druggable targets for *A. baumannii*^10^. Recently, proteome based approaches are used to develop subunit vaccine^73^.

In the present study, we have used comparative proteomes of 52 different strains of *A. baumannii* to identify their shared and unique features such as essential proteins, resistant determinants, and virulent proteins. Identified membrane proteins with the role in virulence and essential survival of *A. baumannii*, were selected for development of vaccine construction. The immune system reacts to some of foreign molecules with the help of T and B cells and generates efficient immune response against pathogens. Interestingly, this immune response has been replicated with synthetic peptides or epitopes against various infectious organisms, including ESKAPE pathogen like *A. baumannii*. Promiscuous epitopes were designed against MHC-class-I, II alleles and B cells, using protein sequence of selected two membrane proteins i.e. channel-tunnel spanning the outer membrane periplasm segregation of daughter chromosomes proteins and penicillin binding protein 1B. Only those peptides, having 100% binding affinity towards all the HLA alleles and IC_50_ values < 200 nM were considered as promiscuous peptides. Predicted peptides were analysed for allergenicity, antigenicity, and solubility, physiochemical properties, etc. Final vaccine constructs were designed with the help of different adjuvants and amino acids linkers. Furthermore, docking analysis was done to explore the binding affinity of promiscuous epitopes with the different HLA molecules i.e. DRB1*0101, DRB3*0202, DRB5*0101, DRB3*0101, DRB1*0401, and DRB1*0301. To analyse the effect of adjuvant and enhance the immune response against vaccine construct, we have also checked interaction between vaccine construct V4 with the TL4/MD2 complex. In the present study, promiscuous peptides predicted, and analysis of promising epitopes by using *in-silico* tools are unique peptides to best of our knowledge. Chimeric subunit vaccine development against the *A. baumannii* may use to generate the immune response against all the finalized epitopes (epitopes of chimeric peptides of finalised proteins) and potential to bind with more than one HLA alleles. Therefore, the present study will help to develop a suitable therapeutics against *A. baumannii* and may help to reduce the mortality and morbidity caused by its infection. In addition to the therapeutic use of the vaccine, people in intensive care units or in hospitals setup can be vaccinated before admission in order to protect them from this nosocomial pathogen. In the vaccine construct, we have added adaptor beta-defensin, Pan-DR epitopes, linker along with multi-epitope sequences that may enhance the significant *A. baumannii* specific immune responses. It is reported that PADRE sequence will reduce the polymorphism of HLA DR molecules in the population^54^. In the murine model, vaccine showed the very high CTL responses than the vaccines without Pan-DR sequence^64^. Murine beta-defensin act as ligands for TLR4 to promote the maturation of dendrite cells^74,75^. In addition, with G-rich linkers in vaccine enhanced the immunogenicity of the multi-subunit vaccine constructs^55^. Therefore, we have taken all important factors that can induce the immunogenicity of our vaccine constructs.

## Conclusion and future prospects

In the present study, we have attempted two approaches subtractive proteomics and reverse vaccinology approach to finding out the suitable non-human homologous targets for the development of vaccine construct. Selected vaccine targets proteins were used to develop the chimeric subunit vaccine. This study started with the retrieval of all *A. baumannii* strains and shortlisted them according to their redundancy and non-redundancy. BLAST of non-redundant strains was performed with respect to reference proteome and identified the shared proteins. All proteins were shortlisted on the basis of their essentiality, virulence factors, resistance gene, non-human homologous proteins and druggability analysis. After shortlisted, thirteen proteins were screened as suitable druggable targets. Out of which four proteins were present on the membrane and two proteins were identified as suitable vaccine antigenic targets. This step was followed by the prediction of immunogenic B-cell and T-cell epitope to generate the humoral and cell-mediated immunity, respectively. Predicted epitopes were merged using suitable linkers and adjuvant to enhance the immunogenicity and effective separation of epitopes within the human body. Allergenicity, conservancy, toxicity, antigenicity and secondary structure analysis were also confirmed followed by the physiochemical properties evaluation. Molecular docking was also performed to check the binding affinity and stability of HLA molecules and TLR-4 with respect to vaccine complex. At last, *in-silico* cloning was performed to ensure the stability and effective expression of vaccine construct (V4). Furthermore, the proposed vaccine needs to be experimentally validated to ensure its use to control *A. baumannii* infections by effective immunological memory. Our predicted *in-silico* results were based on diligent analysis of sequence and various immune databases. *In-silico* studies save both time and costs for researchers and can guide the experimental work, with higher probabilities of finding the desired solutions and with fewer trial and error repeats of assays. The proposed vaccine candidates need a validation in animal models.

## Electronic supplementary material

Supplementary Information

## References

[1] Roy, R., Tiwari, M., Donelli, G. & Tiwari, V. Strategies for combating bacterial biofilms: A focus on anti-biofilm agents and their mechanisms of action. *Virulence*, 10.1080/21505594.21502017.21313372,10.1080/21505594.2017.1313372 (2017). 10.1080/21505594.2017.1313372 PMC5955472 28362216

[2] Tiwari V, Moganty RR (2014). Conformational stability of OXA-51 beta-lactamase explains its role in carbapenem resistance of Acinetobacter baumannii. J Biomol Struct Dyn.

[3] Tiwari V, Rajeswari MR (2013). Effect of Iron Availability on the Survival of Carbapenem-Resistant Acinetobacter baumannii: a Proteomic Approach. Journal of Proteomics & Bioinformatics.

[4] Tiwari V, Vashistt J, Kapil A, Moganty RR (2012). Comparative proteomics of inner membrane fraction from carbapenem-resistant Acinetobacter baumannii with a reference strain. PloS one.

[5] Tiwari V, Nagpal I, Subbarao N, Moganty RR (2012). *In-silico* modeling of a novel OXA-51 from beta-lactam-resistant Acinetobacter baumannii and its interaction with various antibiotics. Journal of molecular modeling.

[6] Tiwari V, Kapil A, Moganty RR (2012). Carbapenem-hydrolyzing oxacillinase in high resistant strains of Acinetobacter baumannii isolated from India. Microb Pathog.

[7] Gonzalez-Villoria AM, Valverde-Garduno V (2016). Antibiotic-Resistant Acinetobacter baumannii Increasing Success Remains a Challenge as a Nosocomial Pathogen. Journal of pathogens.

[8] Badmasti F (2015). Immunological evaluation of OMV(PagL)+Bap(1-487aa) and AbOmpA(8-346aa)+Bap(1-487aa) as vaccine candidates against Acinetobacter baumannii sepsis infection. Molecular immunology.

[9] Ahmad TA, Tawfik DM, Sheweita SA, Haroun M, El-Sayed LH (2016). Development of immunization trials against Acinetobacter baumannii. Trials in Vaccinology.

[10] Kaur N (2013). Identification of druggable targets for Acinetobacter baumannii via subtractive genomics and plausible inhibitors for MurA and MurB. Applied biochemistry and biotechnology.

[11] Barh D, Misra AN, Kumar A, Vasco A (2010). A novel strategy of epitope design in Neisseria gonorrhoeae. Bioinformation.

[12] Zheng, J. *et al*. In Silico Analysis of Epitope-Based Vaccine Candidates against Hepatitis B Virus Polymerase Protein. *Viruses* **9**, 10.3390/v9050112 (2017). 10.3390/v9050112 PMC5454424 28509875

[13] Kumar Jaiswal, A. *et al*. An In Silico Identification of Common Putative Vaccine Candidates against Treponema pallidum: A Reverse Vaccinology and Subtractive Genomics Based Approach. *International journal of molecular sciences* **18**, 10.3390/ijms18020402 (2017). 10.3390/ijms18020402 PMC5343936 28216574

[14] Lee NH (2012). A review of vaccine development and research for industry animals in Korea. Clin Exp Vaccine Res.

[15] Mondal SI (2015). Identification of potential drug targets by subtractive genome analysis of Escherichia coli O157:H7: an in silico approach. Advances and applications in bioinformatics and chemistry: AABC.

[16] Hasan MA, Khan MA, Sharmin T, Hasan Mazumder MH, Chowdhury AS (2016). Identification of putative drug targets in Vancomycin-resistant Staphylococcus aureus (VRSA) using computer aided protein data analysis. Gene.

[17] Dutta A (2006). In silico identification of potential therapeutic targets in the human pathogen Helicobacter pylori. In silico biology.

[18] Lagesen K (2007). RNAmmer: consistent and rapid annotation of ribosomal RNA genes. Nucleic acids research.

[19] Tamura K, Stecher G, Peterson D, Filipski A, Kumar S (2013). MEGA6: Molecular Evolutionary Genetics Analysis version 6.0. Mol Biol Evol.

[20] Vesth T, Lagesen K, Acar O, Ussery D (2013). CMG-biotools, a free workbench for basic comparative microbial genomics. PloS one.

[21] Huang Y, Niu B, Gao Y, Fu L, Li W (2010). CD-HIT Suite: a web server for clustering and comparing biological sequences. Bioinformatics.

[22] Gao F, Luo H, Zhang CT, Zhang R (2015). Gene essentiality analysis based on DEG 10, an updated database of essential genes. Methods in molecular biology (Clifton, N.J.).

[23] Chen L (2005). VFDB: a reference database for bacterial virulence factors. Nucleic acids research.

[24] Gupta SK (2014). ARG-ANNOT, a new bioinformatic tool to discover antibiotic resistance genes in bacterial genomes. Antimicrobial agents and chemotherapy.

[25] Pourhajibagher M, Bahador A (2016). Designing and in Silico Analysis of PorB Protein from Chlamydia Trachomatis for Developing a Vaccine Candidate. Drug research.

[26] Kanehisa M, Goto S (2000). KEGG: kyoto encyclopedia of genes and genomes. Nucleic acids research.

[27] Knox C (2011). DrugBank 3.0: a comprehensive resource for ‘omics’ research on drugs. Nucleic acids research.

[28] Yu NY (2010). PSORTb 3.0: improved protein subcellular localization prediction with refined localization subcategories and predictive capabilities for all prokaryotes. Bioinformatics.

[29] Doytchinova IA, Flower DR (2007). VaxiJen: a server for prediction of protective antigens, tumour antigens and subunit vaccines. BMC bioinformatics.

[30] Larsen MV (2007). Large-scale validation of methods for cytotoxic T-lymphocyte epitope prediction. BMC bioinformatics.

[31] Kim Y (2012). Immune epitope database analysis resource. Nucleic acids research.

[32] Nielsen M (2003). Reliable prediction of T-cell epitopes using neural networks with novel sequence representations. Protein science: a publication of the Protein Society.

[33] Peters B (2005). The immune epitope database and analysis resource: from vision to blueprint. PLoS biology.

[34] Sidney J (2008). Quantitative peptide binding motifs for 19 human and mouse MHC class I molecules derived using positional scanning combinatorial peptide libraries. Immunome research.

[35] Lundegaard C (2008). NetMHC-3.0: accurate web accessible predictions of human, mouse and monkey MHC class I affinities for peptides of length 8-11. Nucleic acids research.

[36] Momtaz S, Rahman A, Sultana M, Hossain MA (2014). Evolutionary Analysis and Prediction of Peptide Vaccine Candidates for Foot-and-Mouth-Disease Virus Types A and O in Bangladesh. Evolutionary bioinformatics online.

[37] Calis JJ (2013). Properties of MHC class I presented peptides that enhance immunogenicity. PLoS computational biology.

[38] Bui HH, Sidney J, Li W, Fusseder N, Sette A (2007). Development of an epitope conservancy analysis tool to facilitate the design of epitope-based diagnostics and vaccines. BMC bioinformatics.

[39] Dash R (2017). In silico-based vaccine design against Ebola virus glycoprotein. Advances and applications in bioinformatics and chemistry: AABC.

[40] Gupta S (2013). In silico approach for predicting toxicity of peptides and proteins. PloS one.

[41] Wang P (2008). A systematic assessment of MHC class II peptide binding predictions and evaluation of a consensus approach. PLoS computational biology.

[42] Wang P (2010). Peptide binding predictions for HLA DR, DP and DQ molecules. BMC bioinformatics.

[43] Thomsen M, Lundegaard C, Buus S, Lund O, Nielsen M (2013). MHCcluster, a method for functional clustering of MHC molecules. Immunogenetics.

[44] El-Manzalawy Y, Dobbs D, Honavar V (2008). Predicting linear B-cell epitopes using string kernels. Journal of molecular recognition: JMR.

[45] El-Manzalawy Y, Dobbs D, Honavar V (2008). Predicting flexible length linear B-cell epitopes. Computational systems bioinformatics. Computational Systems Bioinformatics Conference.

[46] Ponomarenko JV, Bourne PE (2007). Antibody-protein interactions: benchmark datasets and prediction tools evaluation. BMC structural biology.

[47] Karplus PA, Schulz GE (1985). Prediction of chain flexibility in proteins. Naturwissenschaften.

[48] Chou PY, Fasman GD (1978). Prediction of the secondary structure of proteins from their amino acid sequence. Advances in enzymology and related areas of molecular biology.

[49] Kolaskar AS, Tongaonkar PC (1990). A semi-empirical method for prediction of antigenic determinants on protein antigens. FEBS letters.

[50] Emini EA, Hughes JV, Perlow DS, Boger J (1985). Induction of hepatitis A virus-neutralizing antibody by a virus-specific synthetic peptide. Journal of virology.

[51] Parker JM, Guo D, Hodges RS (1986). New hydrophilicity scale derived from high-performance liquid chromatography peptide retention data: correlation of predicted surface residues with antigenicity and X-ray-derived accessible sites. Biochemistry.

[52] Ponomarenko J (2008). ElliPro: a new structure-based tool for the prediction of antibody epitopes. BMC bioinformatics.

[53] Rana A, Akhter Y (2016). A multi-subunit based, thermodynamically stable model vaccine using combined immunoinformatics and protein structure based approach. Immunobiology.

[54] Ghaffari-Nazari H (2015). Improving Multi-Epitope Long Peptide Vaccine Potency by Using a Strategy that Enhances CD4+T Help in BALB/c Mice. PloS one.

[55] Yang Y (2015). In silico design of a DNA-based HIV-1 multi-epitope vaccine for Chinese populations. Human Vaccines & Immunotherapeutics.

[56] Saha S, Raghava GP (2006). AlgPred: prediction of allergenic proteins and mapping of IgE epitopes. Nucleic acids research.

[57] Magnan CN (2010). High-throughput prediction of protein antigenicity using protein microarray data. Bioinformatics.

[58] Magnan CN, Randall A, Baldi P (2009). SOLpro: accurate sequence-based prediction of protein solubility. Bioinformatics.

[59] Kosciolek T, Jones DT (2014). De novo structure prediction of globular proteins aided by sequence variation-derived contacts. PloS one.

[60] Tiwari, V., Tiwari, M. & Biswas, D. Rationale and design of an inhibitor of RecA protein as an inhibitor of Acinetobacter baumannii. *The Journal of Antibiotics*, 10.1038/s41429-018-0026-2 (2018). 10.1038/s41429-018-0026-2 29410519

[61] Grote A (2005). JCat: a novel tool to adapt codon usage of a target gene to its potential expression host. Nucleic acids research.

[62] Mori H, Maruyama F, Kurokawa K (2010). VITCOMIC: visualization tool for taxonomic compositions of microbial communities based on 16S rRNA gene sequences. BMC bioinformatics.

[63] Oany AR (2017). Vaccinomics Approach for Designing Potential Peptide Vaccine by Targeting Shigella spp. Serine Protease Autotransporter Subfamily Protein SigA..

[64] Wu CY, Monie A, Pang X, Hung CF, Wu TC (2010). Improving therapeutic HPV peptide-based vaccine potency by enhancing CD4+T help and dendritic cell activation. J Biomed Sci.

[65] Kashyap M, Jaiswal V, Farooq U (2017). Prediction and analysis of promiscuous T cell-epitopes derived from the vaccine candidate antigens of Leishmania donovani binding to MHC class-II alleles using in silico approach. Infection, genetics and evolution: journal of molecular epidemiology and evolutionary genetics in infectious diseases.

[66] Tiwari V, Tiwari D, Patel V, Tiwari M (2017). Effect of secondary metabolite of Actinidia deliciosa on the biofilm and extra-cellular matrix components of Acinetobacter baumannii. Microb Pathog.

[67] Tiwari, V., Roy, R. & Tiwari, M. Antimicrobial active herbal compounds against Acinetobacter baumannii and other pathogens. *Frontiers in Microbiology* **6**, 10.3389/fmicb.2015.00618 (2015). 10.3389/fmicb.2015.00618 PMC4471432 26150810

[68] Tiwari M, Roy R, Tiwari V (2016). Screening of Herbal-Based Bioactive Extract Against Carbapenem-Resistant Strain of Acinetobacter baumannii. Microbial Drug Resistance.

[69] Tiwari V, Raghav R, Tiwari M (2015). Comparative anti-bacterial activity of differently capped silver nanomaterial on the carbapenem sensitive and resistant strains of Acinetobacter baumannii. Journal of Nanomedicine & Nanotechnology.

[70] Tiwari V, Tiwari M, Solanki V (2017). Polyvinylpyrrolidone-Capped Silver Nanoparticle Inhibits Infection of Carbapenem-Resistant Strain of Acinetobacter baumannii in the Human Pulmonary EpithelialCell. Front. Immunol..

[71] Tiwari, V., Patel, V. & Tiwari, M. In-silico screening and experimental validation reveal L-Adrenaline as anti-biofilm molecule against biofilm-associated protein (Bap) producing Acinetobacter baumannii. *International Journal of Biological Macromolecules*, 10.1016/j.ijbiomac.2017.09.105 (2017). 10.1016/j.ijbiomac.2017.09.105 28964839

[72] Verma, P., Tiwari, M. & Tiwari, V. In-silico High Throughput Virtual Screening and Molecular Dynamics Simulation study to identify Inhibitor for AdeABC Efflux Pump of Acinetobacter baumannii. *J Biomol Struct Dyn*, 10.1080/07391102.07392017.01317025, 10.1080/21505594.2017.1313372 (2017). 10.1080/07391102.2017.1317025 28393677

[73] Moriel DG (2013). Identification of novel vaccine candidates against multidrug-resistant Acinetobacter baumannii. PloS one.

[74] Mei HF (2012). beta-defensin 2 as an adjuvant promotes anti-melanoma immune responses and inhibits the growth of implanted murine melanoma *in vivo*. PloS one.

[75] Biragyn A (2002). Toll-like receptor 4-dependent activation of dendritic cells by beta-defensin 2. Science.

