# Supplementary material for: Subtractive proteomics to identify novel drug targets and reverse vaccinology for the development of chimeric vaccine against Acinetobacter baumannii
Source: Sci Rep. 2018 Jun 13;8:9044. doi: 10.1038/s41598-018-26689-7 (PMC5997985; doi:10.1038/s41598-018-26689-7)
Supplement: Supplementary file 1 — Supplementary Information [file 41598_2018_26689_MOESM1_ESM.pdf]

Supplementary Tables and Supplementary Figures

Title: Subtractive genomics to identify novel drug targets and reverse vaccinology for the development of chimeric vaccine against *Acinetobacter baumannii*

Authors: Vandana Solanki and Vishvanath Tiwari*

Table ST-1: Genome information of all 52 strains of *Acinetobacter baumannii* used in phylogenic analysis.

S.N.	Uniport ID	Organism	INSDC	WGS	WGS for download	Status
1	UP000002446	*A. baumannii* AYE	CU459141.1 CU459140.1 CU459139.1 CU459138.1 CU459137.1	-	-	complete
2	UP000002844	*A. baumannii* Naval-82	-	AMSW01	AMSW00000000	Contig
3	UP000005740	*A. baumannii* (strain ATCC 19606 / (Strain: CIP 70.34)	-	JMRY01	JMRY00000000	Scaffold
4	UP000009290	*A. baumannii* MDR-ZJ06	CP001937.1 CP001938.1	-	-	Complete
5	UP000011533	*A. baumannii* WC-A-92	-	AMFU01	-	Contig
6	UP000011642	*A. baumannii* Naval-57	-	AMFP01	AMFP00000000	contig
7	UP000013021	*A. baumannii* NIPH 80	-	APRE01	APRE00000000	Scaffold
8	UP000013023	*A. baumannii* NIPH 201	-	APQV01	APQV00000000	Scaffold
9	UP000013068	*A. baumannii* NIPH 601	-	APQZ01	APQZ00000000	Scaffold
10	UP000013132	*A. baumannii* ATCC 19606 = CIP 70.34 = JCM 6841	-	APRG01	APRG00000000	Scaffold
11	UP000013284	*A. baumannii* NIPH 190	-	APPL01	APPL00000000	scafold
12	UP000013288	*A. baumannii* NIPH 60	-	APPM01	APPM00000000	Scaffold
13	UP000014561	*A. baumannii* NIPH 410	-	ATGJ01	ATGJ00000000	Scaffold
14	UP000016517	*A. baumannii* EGD-HP18	-	AVST01	AVST00000000	Contig
15	UP000016884	*A. baumannii* NCGM 237	AP013357.1	-	-	complete
16	UP000020489	*A. baumannii* 1437282	-	JEWN01	JEWN00000000	Contig
17	UP000020595	*A. baumannii* 1295743	-	JEWH01	JEWH00000000	Contig
18	UP000020632	*A. baumannii* 951631	-	JEXI01	JEXI00000000	Contig
19	UP000020680	*A. baumannii* 348935	-	JEVW01	JEVW00000000	Contig
20	UP000020735	*A. baumannii* 99063	-	JEXJ01	JEXJ00000000	Contig
21	UP000021108	*A. baumannii* 625974	-	JEXD01	JEXD00000000	Contig
22	UP000021295	*A. baumannii* 118362	-	JEWB01	JEWB00000000	Contig
23	UP000021421	*A. baumannii* 1419130	-	JEWL01	JEWL00000000	Contig
24	UP000021741	*A. baumannii* 1000160	-	JSUR01	JSUR00000000	Contig
25	UP000022150	*A. baumannii* 573719	-	JFYA01	JFYA00000000	Contig
26	UP000023317	*A. baumannii* 146457	-	JEWS01	JEWS00000000	Contig
27	UP000027060	*A. baumannii* 233846	-	JMOG01	JMOG00000000	Contig
28	UP000027078	*A. baumannii* 1571545	-	JMOM01	JMOM00000000	Contig
29	UP000027130	*A. baumannii* 1288284	-	JMOF01	JMOF00000000	Contig

30	UP000027155	*A. baumannii* 754286	-	JMNU01	JMNU00000000	Contig
31	UP000027233	*A. baumannii* 496487	-	JMNR01	JMNR00000000	Contig
32	UP000027254	*A. baumannii* 15827	-	JMNX01	JMNX00000000	Contig
33	UP000027276	*A. baumannii* 25977_9	-	JMPF01	JMPF00000000	Contig
34	UP000027319	*A. baumannii* 940793	-	JMNW01	JMNW00000000	Contig
35	UP000027327	*A. baumannii* 21072	-	JMOD01	JMOD00000000	Contig
36	UP000027334	*A. baumannii* 855125	-	JMNT01	JMNT00000000	Contig
37	UP000028932	*A. baumannii* AB30	CP009257.1	-	-	Complete
38	UP000031035	*A. baumannii* 1	-	JSAO01,<br>JSUR01	JSAO00000000,	Contig
39	UP000032746	*A. baumannii* AB5075-UW	CP008706.1<br>CP008707.1<br>CP008708.1<br>CP008709.1	-	-	Complete
40	UP000036122	*A. baumannii* MRSN 3527	-	JPHZ01	JPHZ00000000	contig
41	UP000036720	*A. baumannii* B8342	CP021342.1	-	-	complete
42	UP000037552	*A. baumannii* SP1917	-	LFYW01	LFYW00000000	contig
43	UP000050896	*A. baumannii* ABBL070	-	LLGA01	LLGA00000000	contig
44	UP000051322	*A. baumannii* ABBL059	-	LLFE01	LLFE00000000	Contig
45	UP000070590	*A. baumannii* XH858	CP014528.1	-	-	Complete
46	UP000076314	*A. baumannii* AB3638	-	LRDW01	LRDW00000000	Scaffold
47	UP000094982	*A. baumannii* XH198	-	MDWM01	MDWM00000000	Scaffold
48	UP000179770	*A. baumannii* XH694	-	LYIX01	LYIX00000000	Scaffold
49	UP000179791	*A. baumannii* XH639	-	LYKQ01	LYKQ00000000	Scaffold
50	UP000179937	*A. baumannii* XH647	-	LYKI01	LYKI00000000	Scaffold
51	UP000188718	*A. baumannii* ATCC 17945	-	MTGG01	MTGG00000000	Scaffold
52	UP000001741	*A. baumannii* (strain SDF)	CU468230.2<br>CU468231.2<br>CU468232.2<br>CU468233.2	-	-	Chromosome

Table ST2: List of KEGG database unique pathways present in *A. baumannii* with respect to human

S.No	Metabolic pathway dependent proteins	Number of DEG proteins	Number of VFDB proteins	Number of ARG proteins
**UNIQUE PATHWAYS**				
1	01110 Biosynthesis of secondary metabolities	55	6	-
2	01120 microbial metabolism in diverse environment	19	4	-
3	01130 biosynthesis of antibiotics	36	2	-
4	01220degradation of aromatic compounds	-	1	-
5	00660 C5 branched dibasic acid metabolism	1	-	-
6	00680 methane metabolism	2	-	-
7	00300 lysine biosynthesis	4	-	-
8	00540 lipopolysaccharide biosynthesis	8	5	-
9	00550 peptidoglycan biosynthesis	-	-	1
10	00281 Geraniol degradation	-	1	-
11	00261 Monobactam biosynthesis	2	-	-
12	00401 Novobiocin biosynthesis	1	-	-
13	00625 Chloroalkane and chloroalkene degradation	-	1	-
14	00626 Naphthalene degradation	-	1	-
15	02060 Phosphotransferase system (PTS)	1	-	-
16	03070 Bacterial secretion system	5	8	-
17	02020 Two-component system	2	14	-
18	02024 Quorum sensing	1	4	-
19	01501 beta-Lactam resistance	1	3	2
20	01502 Vancomycin resistance	7	-	-
21	01503 Cationic antimicrobial peptide (CAMP) resistance	-	2	1
22	00460 cyano amino acid metabolism	-	-	-
23	00473 D-alanine metabolism	-	-	-
24	00332 Carbapenem biosynthesis	-	-	-
25	00521 Streptomycin biosynthesis	-	-	-
26	00623 Toluene degradation	-	-	-
27	00622 Xylene degradation	-	-	-
28	00643 Styrene degradation	-	-	-
29	00930 Caprolactam degradation	-	-	-
30	00362 Benzoate degradation	-	-	-
31	00364 Fluorobenzoate degradation	-	-	-
32	00903 Limonene and pinene degradation	-	-	-
33	00361 Chlorocyclohexane and chlorobenzene degradation	-	-	-

Table ST3: Antigenicity prediction of potential protein targets using VaxiJen web server.

Uniport ID	NAME	Potent Antigenicity
B0VUZ6	Penicillin-binding protein 1B	0.5541
B0VMD0	Channel-tunnel spanning the outer membrane and periplasm segregation of daughter chromosomes	0.5789
B0VTR5	PutativeD-ala-D-ala-carboxypeptidase, penicillin-binding protein	0.4860
B0VT18	Putative Oxidoreductase, short chain dehydrogenase/reductase family	0.3296

Table ST4: Predicted MHC class I epitopes of *A. baumannii* proteins using NetCTL.

S N.	Uniport ID	Protein name	Peptide	**Aff**	**Aff rescale**	**cleavage**	**TAP**	**COMB**
1	B0VMD0	Channel-tunnel spanning the outer membrane and periplasm segregation of daughter chromosomes	TSSSFALDL	0.2133	0.9058	0.8246	0.8650	1.0728
			SSSFALDLV	0.1653	0.7018	0.5187	0.4140	0.8003
			FALDLVETY	0.2338	0.9927	0.9776	2.9540	1.2871
			LSDALVSNT	0.1934	0.8212	0.0490	-.8670	0.7852
			FRMDAWEGY	0.1170	0.4969	0.9311	3.1490	0.7940
			HVLNVAEAY	0.2070	0.8789	0.8962	2.8510	1.1559
			RQQALTAAY	0.1130	0.4799	0.7911	3.2600	0.7616
			DVSEANAQY	0.2559	1.0867	0.9712	2.7970	1.3722
			LAQEQLSEY	0.2289	0.9717	0.9525	2.9970	1.2644
			QLSEYIGPY	0.3921	1.6646	0.9667	2.7860	1.9489
			LTQQQNLKI	0.1688	0.7165	0.7636	0.4550	0.8538
			STDGKFDQV	0.2426	1.0300	0.6322	0.1790	1.1338
			GVEMNWNLF	0.1364	0.5789	0.6443	2.3970	0.7954
			NVDVKSAFM	0.1474	0.6257	0.8882	0.4580	0.7818
			DTDRAKLEA	0.1788	0.7590	0.3825	- 1.0020	0.7663
			VSQASKASY	0.2699	1.1459	0.9422	3.0430	1.4394
			KQDYLNAQY	0.3775	1.6027	0.7448	3.0850	1.8687
2	B0VUZ6	Penicillin-binding protein 1B OS	TQELKLLGY	0.2356	1.0003	0.9085	2.7300	1.2731
			YVAQGSNMY	0.5364	2.2774	0.9707	3.0700	2.5765
			YVHTRGFDY	0.3263	1.3854	0.9559	3.1090	1.6843
			ISTEDRNFY	0.3387	1.4383	0.8931	3.0000	1.7222
			STEDRNFYH	0.3052	1.2957	0.0843	- 0.6390	1.2764
			TQQLVKNFY	0.1735	0.7366	0.7543	2.9950	0.9995
			MALLIELHY	0.1953	0.8291	0.9730	3.0900	1.1296
			SKDEILEAY	0.2228	0.9462	0.8985	2.7990	1.2209
			ELNVAQQAY	0.1723	0.7314	0.9567	2.7780	1.0138
			GLVQGPSLY	0.1313	0.5574	0.9749	2.8900	0.8481
			ESDLTNQGL	0.1453	0.6169	0.9230	0.5660	0.7837
			GSLLKPVIY	0.1545	0.6558	0.9600	2.8510	0.9424
			LSAIESGRY	0.6710	2.8491	0.8506	2.9340	3.1234
			LSEALANSY	0.7008	2.9757	0.8857	2.8790	3.2525
			STFTNNLRK	0.1465	0.6219	0.9392	0.6560	0.7955
			GVESTIPAY	0.2100	0.8914	0.7252	2.6350	1.1320
			VVDANGHLL	0.1820	0.7727	0.8466	1.0330	0.9513
			LLDRYGLNV	0.2126	0.9026	0.9702	0.1390	1.0550
			ETIDPSVGY	0.1659	0.7042	0.9324	2.7190	0.9800
			PSVGYIMNY	0.2085	0.8853	0.9741	2.4240	1.1526
			SSGTGRAAY	0.2798	1.1879	0.9405	2.8910	1.4735
			TRDSWFAGY	0.2431	1.0320	0.9780	2.7950	1.3185
			WIDHASGDL	0.1695	0.7198	0.9275	0.8310	0.9004
			AQACDGAMY	0.1759	0.7470	0.3208	3.2150	0.9559
			RATPCGAPY	0.1076	0.4570	0.9192	3.1620	0.7530
			ATPCGAPYY	0.2794	1.1861	0.9763	3.1640	1.4908
			PEDDNTDSY	0.1311	0.5567	0.9598	1.9660	0.7990

Table ST5: Predicted MHC I epitopes, HLA alleles interaction and class I immunogenicity analysis using IEDB server.

S.No	Protein name	Peptide	Interacting MHC I Alleles (IEDB)	Class I Immunogenicity
1	B0VMD0<br>Channel-tunnel spanning the outer membrane and periplasm segregation of daughter chromosomes	DVSEANAQY	HLA-A*26:01(0.2)	0.044
		SSSFALDLV	HLA-A*68:02(1.6)	0.104
		STDGKFDQV	HLA-A*02:06(2.05)	-0.10746
		FALDLVETY	HLA-B*53:01(0.2), HLA-B*35:01(0.2)	0.15396
		HVLNVAEAY	HLA-B*35:01(0.5) HLA-A*30:02(1.1)	0.17428
		RQQALTAAY	HLA-B*15:01(0.1), HLA-A*30:02(0.15) HLA-A*32:01(1.7)	0.08339
		LAQEQLSEY	HLA-B*35:01(0.4)	-0.14121
		QLSEYIGPY	HLA-A*26:01 (0.5) HLA-B*15:01(0.5)	0.19085
		VSQASKASY	HLA-B*15:01 (0.9) HLA-A*30:02(0.25)	-0.42597
		KQDYLNAQY	HLA-A*30:02(0.2)	-0.04807
2	B0VUZ6<br>Penicillin-binding protein 1B OS	YVAQGSNMY	HLA-A*26:01(0.2), HLA-B*35:01(0.3), HLA-B*15:01(0.5), HLA-A*30:02(0.55)	-0.33465
		MALLIELHY	HLA-B*53:01(0.3), HLA-B*35:01(0.5), HLA-B*58:01(0.6), HLA-B*57:01(0.75)	0.21863
		LSAIESGRY	HLA-A*30:02(0.2), HLA-A*01:01(0.25)	0.14723
		LSEALANSY	HLA-A*01:01(0.2), HLA-A*30:02(1), HLA-B*35:01(2.6)	-0.00422
		STFTNNLRK	HLA-A*11:01(0.2), HLA-A*03:01(0.3), HLA-A*68:01(0.5), HLA-A*30:01(0.8)	0.08555
		LLDRYGLNV	HLA-A*02:01(1.3), HLA-A*02:03(1.85) HLA-A*02:06(2.55)	0.0744
		ETIDPSVGY	HLA-A*26:01(0.1), HLA-A*68:01(1), HLA-A*68:02(1.9)	-0.04637
		SSGTGRAAY	HLA-A*30:02(0.25), HLA-B*15:01(1.2)	0.18766
		GVESTIPAY	HLA-A*30:02(0.85)	0.04261
		TQQLVKNFY	HLA-A*30:02(0.85)	-0.14862
		ISTEDRNFY	HLA-A*30:02(0.55)	0.24661
		YVHTRGFDY	HLA-B*35:01(1.7)	0.24362

Table ST6: Predicted B cell epitopes of protein B0VMD0 using four different tools

SN.	Position	Final Epitope (B0VMD0)	FBCpred (score)	BCpred (score)	Eillipro (score)	IEDB server (Bepipred server) (Mini-Max score)
1	62-106	VTLSGNITRNRQTVKRSNFPGVDQEGLSDALVSNTSTTKQATLSA	VTLSGNITRNRQ (0.995) SDALVSNTSTTKQA (0.087)	SDALVSNTSTTKQATLSARQ(.748) ITRNRQTVKRSNFPGVDQEG (0.942)	VTLSGNITRNRQTVKRSNFPGVDQEGLSDALVSNTSTTKQATLSA (0.851)	TLSGNITRNRQTVKRSNFPGVDQEGLSDALVSNTSTTKQATLSA (0.056-1.208)
2	162-204	LQEEKALLEQLNMMNAKLKEGLVARSDVSEANQYQNARANRI	LQEEKALLEQLN (0.998) MNAKLKEGLVARSD(0.943)	-	LQEEKALLEQLNMMNAKLKEGLVARSDVSEANQYQNARANRI (0.804)	VARSDVSEANAQYQNARANRI (0.091-1.130)
3	231-335	LRSDFIFQKPYPAQLDEWLGLTQQQNLKIQQARLQKRYAEDQRRVEKEKAALYPQIDAVASYGYTKQTPETLISTDGKFDQGVEMNWNLFNGGRTRTSIKKASVEL	GLTQQQNLKIQQAR (0.978) FIFQKPYPAQLDEW (0.881) QKRYAEDQRRVEK (0.949) MNWNLFNGGRTRTSIKKAS(0.948) YPQIDAVASYGYTK (0.807)	LRSDFIFQKPYPAQLDEWLG(0.808) KIQQARLQKRYAEDQRRVEK(0.93) EMNWNLFNGGRTRTSIKKAS(0.905) VASYGYTKQTPETLISTDGK(0.891)	LRSDFIFQKPYPAQLDEWLGLTQQQ (0.596) EKAALYPQIDAVASYGYTKQTPETLISTDGKFDQVGVEMNWNLFNGGRTRTSIKKASVELN(0.0715)	LQKRYAEDQRRVEK (0.022-0.917) ALYPQIDAVASYGYTKQTPETLISTDGKFDQVG (-0.000- 1.495) GGRTRTSIKK (0.285-0.846)
4	359-419	QVDTDRAKLEARKAAMDSSALVSQASKASYNEGLKSMVDVLLAQRNAFSAKQDYLNAQYDYL	ASKASYNEGLKSMV (0.872) QVDTDRAKLEARK (0.931) RNAFSAKQDYLNAQ (0.826)		LVSQASKASYNEGLKSMVDVLL (0.685)	SALVSQASKASYNEGLK (0.033-1.096) QVDTDRAKLEARKAAMD (0.077-0.700) AFSAKQDYLNA (0.018-0.645)

Table ST7: Predicted B cell epitopes of protein B0VUZ6 using four different tools

S. N o	Posi tion	Final Epitope (B0VUZ6)	FBCpred	BCpred	Eillipro	IEDB(Bepipred result)
1	688-787	TPVNLRQPDSVQWQWIDHASGDLSAQACDGAMYIPMLAHTVPHRATPCGAPYYQVDPTYTPQSDNTIPEPEDDNTDSYIRESENQMEQDLSNNTRIISSG	ENQMEQDLSNNTRI(1) IPEPEDDNTDSYIR(1) ATPCGAPYYQVDPT(0.999) DHASGDLSAQACDG(0.987) TPVNLRQPDSVQWQ(0.92)	TPQSDNTIPEPEDDNTDSYI(1) AHTVPHRATPCGAPYYQVDP(0.998) ESENQMEQDLSNNTRIISSG (0.995)	LRQPDSVQWQWIDHASGDLSAQACDGAMYIPMLAHTVPHRATPCG(0.794)	RQTPVNLRQPDSVQW (0.058-1.031) IDHASGDLSAQACD (0.061-0.834) VPHRATPCGAPYYQVDPTYTPQSDNTIPEPEDDNTDSYIRESENQMEQDLSNNTRIISS (-0.102-2.701)
2	464-506	SAIESGRYNWASQIEDAPISVPVDGGKSWTPKNYSGGGHGIVS	SWTPKNYSGGGHGI(1), IESGRYNWASQIED(0.866)	VPVDGGKSWTPKNYSGGGHG (.098)	SAIESGRYNWASQIEDAPISVPVDGGKSWTPKNYSGGGHGIVS(0.758)	ESGRYNWASQIEDAPISVPVDGGKSWTPKNYSGGGHGI (0.067-1.781)
3	79-139	KTSSNYDKSGTYVAQGSNMYVHTRGFDYGDSVEPEQVLELSFANDQVVEVRSTKPSSTGVA	GTYVAQGSNMYVHTRGFDYGD (.884) QVVEVRSTKPSSTG(1), FDYGDSVEPEQVLE(0.999)	TYVAQGSNMYVHTRGFDYGD (0.9922) LSFANDQVVEVRSTKPSSTG (0.972)	VAQGSNMYVH(0.734) KTSSNYDK(0.609)DSVEPEQVLELSFANDQVVEVRSTKPSSTGVA(0.717)	KTSSNYDKSGTYVAQGSNMY (0.015-1.503) GFDYGDSVEPEQV (0.154-1.605) VVEVRSTKPSSTGVA(0.245-1.761)
4	174-248	ISTEDRNFYHHHGISIRGTARALVSNVTGGRRQGGSSTLTQQLVKNFYLTPERTLKRKVNEALMALLIELHYSKDE	GGSSTLTQQLVKNF(0.937), TPERTLKRKVNEAL(0.698)	STLTQQLVKNFYLTPERTLK (0.898)	ISTEDRNFYHHHGISIRGTARALVSNVTGGRRQGGSTLTQ(0.762) VKNFYLTPERTLKRKVNEALMALLIELHYSKDE(0.715)	STEDRNF (0.069-0.354) NVTGGRRQGGSTLT (0.047-1.590) ERTLKRK (-0.00-0.599)
6	366-382	RTEYQESDLTNQGLRI	EYQESDLTNQGLRI(0.945)	FLDIVRRQLRTEYQESDLTN (0.952)		RTEYQESDLTN (0.166-1.065)
7	297-325	VQGPSLYNPWKNPEGAKNRRDTVLNNMRV	AKNRRDTVLNNMRV (0.982) VQGPSLYNPWKNPE(.872)	GPSLYNPWKNPEGAKNRRDT (0.974)		GPSLYNPWKNPEGAKNRRDT (0.194-1.961)

Table ST8: Predicted interaction analysis of B cell epitopes with HLA alleles.

SN.	Position	Final B Epitope	MHC II
1	(B0VMD0)<br>62-106	VTLSGNITRNRQTVKRSNFPGVDQEG<br>LSDALVSNTSTTKQATLSA	HLA-DRB1*04:01
2	(B0VMD0)<br>162-204	LQEEKALLEQLNMMNAKLKEGLVAR<br>SDVSEANAQYQNARANRI	HLA-DRB5*01:01<br>HLA-DRB3*02:02<br>HLA-DRB1*07:01<br>HLA-DRB4*01:01
3	(B0VMD0)<br>231-336	LRSDFIFQKPYPAQLDEWLGLTQQQN<br>LKIQQARLQKRYAEDQRRVEKEKAA<br>LYPQIDAVASYGYTKQTPETLISTDGK<br>FDQGVEMNWNLFNGGRTRTSIKKAS<br>VELN	HLA-DRB1*09:01<br>HLA-DRB4*01:01<br>HLA-DRB3*02:02<br>HLA-DRB3*01:01<br>HLA-DQA1*05:01/DQB1*02:01<br>HLA-DQA1*01:02/DQB1*06:02
4	(B0VMD0)<br>359-419	QVDTDRAKLEARKAAMDSSALVSQA<br>SKASYNEGLKSMVDVLLAQRNAFSA<br>KQDYLNAQYDYL	HLA-DRB1*03:01<br>HLA-DRB1*08:02<br>HLA-DRB1*04:01<br>HLA-DRB1*07:01<br>HLA-DQA1*01:02/DQB1*06:02
5	(B0VUZ6) 688-<br>787	TPVNLRQPDSVQWQWIDHASGDLSA<br>QACDGAMYIPMLAHTVPHRATPCGA<br>PYYQVDPTYTPQSDNTIPEPEDDNTDS<br>YIRESENQMEQDLSNNTRIISSG	HLA-DRB4*01:01<br>HLA-DRB1*01:01<br>HLA-DRB3*01:01<br>HLA-DRB5*01:01<br>HLA-DRB1*11:01<br>HLA-DRB1*12:01<br>HLA-DRB1*04:01<br>HLA-DRB3*02:02
6	(B0VUZ6) 79-139	KTSSNYDKSGTYVAQGSNMYVHTRG<br>FDYGDSVEPEQVLELSFANDQVVEVR<br>STKPSSTGVA	HLA-DRB1*07:01<br>HLA-DRB1*04:01<br>HLA-DRB1*12:01<br>HLA-DRB1*08:02<br>HLA-DRB1*03:01<br>HLA-DQA1*01:01/DQB1*05:01<br>HLA-DRB3*01:01
7	(B0VUZ6) 174-<br>248	ISTEDRNFYHHHGISIRGTARALVSNV<br>TGGRRQGGSSTLTQQLVKNFYLTPER<br>TLKRKVNEALMALLIELHYSKDE	HLA-DRB1*08:02<br>HLA-DRB1*07:01<br>HLA-DRB1*03:01<br>HLA-DRB1*13:02<br>HLA-DRB5*01:01<br>HLA-DRB1*07:01<br>HLA-DRB1*09:01<br>HLA-DRB3*02:02<br>HLA-DQA1*04:01/DQB1*04:02<br>HLA-DPA1*02:01/DPB1*01:01<br>HLA-DQA1*03:01/DQB1*03:02<br>HLA-DQA1*05:01/DQB1*03:01<br>HLA-DQA1*01:02/DQB1*06:02
8	(B0VUZ6) 636-<br>658	KLAGKSGTTNDTRDSWFAGYSGN	HLA-DRB1*15:01
9	(B0VUZ6) 366-<br>382	RTEYQESDLTNQGLRI	HLA-DRB1*04:01<br>HLA-DRB1*08:02<br>HLA-DRB1*09:01

Table ST9: Comparative analysis of all predicted B cell, MHC I and MHC II epitopes of protein B0VMD0.

SN.	Position	Final B Epitope (B0VMD0)	MHC II	MHC I
1.	62-106	VTLSGNITRNRQTVKRS NFPGVDQEGLSDALVSN TSTTKQATLSA	-	-
2.	162-204	LQEEKALLEQLNMMNA KLKEGLVARSDVSEANA QYQNARANRI	LEQLNMMNAKLKE GL	DVSEANAQY
3.	231-336	LRSDFIFQKPYPAQLDEW LGLTQQQNLKIQQARLQ KRYAEDQRRVEKEKAA LYPQIDAVASYGYTKQT PETLISTDGKFDQGVEM NWNLFNGGRTRTSIKKA SVELN	-	-
4.	359-419	QVDTDRAKLEARKAAM DSSALVSQASKASYNEG LKSMVDVLLAQRNAFSA KQDYLNAQYDYL	MVDVLLAQRNAFS AK	-
5.	-	-	-	SSSFALDLV

Table ST10: Comparative analysis of all predicted B cell, MHC I and MHC II epitopes of protein B0VUZ6.

S.No	Positions	Final Epitopes (B0VUZ6)	MHC II	MHC I
1	688-787	TPVNLRQPDSVQWQWIDHASGDLSAQ ACDGAMYIPMLAHTVPHRATPCGAPY YQVDPTYTPQSDNTIPEPEDDNTDSYI RESENQMEQDLSNNTRIISSG	-	-
2	464-506	SAIESGRYNWASQIEDAPISVPVDGGK SWTPKNYSGGGHGIVS	-	-
3	79-139	KTSSNYDKSGTYVAQGSNMYVHTRG FDYGDSVEPEQVLELSFANDQVVEVR STKPSSTGVA	-	YVHTRGFDY
4	174-248	ISTEDRNFYHHHGISIRGTARALVSNV TGGRRQGGSSTLTQQLVKNFYLTPER TLKRKVNEALMALLIELHYSKDE	STEDRNFYHHHGISI	MALLIELHY ISTEDRNFY
6	366-382	RTEYQESDLTNQGLRI	-	-
7	297-325	VQGPSLYNPWKNPEGAKNRRDTVLN NMRV	-	-
8	624-639	-	GRAAYNSLSPALKLA	-
9	528-543	-	LSTFTNNLRKFGVES	-
10	3-18	-	FERGIGFFALIFSIL	-
11	24-39	-	ALSIYLIRLDNIIRE	-
12	440-455	-	FTGFNRALDAKRQVG	-
13	-	-	-	LLDRYGLNV
14	-	-	-	SSGTGRAAY

Table ST11: Analysis of codon adaptation, antigenicity and solubility analysis of vaccine constructs (V1 to V4).

Name with adjuvant.	Nucleotide	CAI value	GC content	ANTIGENpro	Vaxijen 2.0	SOLpro
V1	1600	0.9824	53.4430	0.951428	0.7959	0.882
V2	1600	0.9675	52.9739	0.944662	0.8007	0.920
V3	1250	0.9883	52.4249	0.953745	0.8636	0.779
V4	1100	1	51.058	0.912087	0.8716	0.797

Table ST12: Biophysical characteristics of vaccine construct (V1 to V4) by ProtParam.

Name with adjuvant	Number of amino acid	Molecular weight (KDa)	PI	Aliphatic index	hydropathicity (GRAVY)	Instability index
V1	547	58.83	4.86	72.61	-0.527	34.93
V2	538	57.71	4.82	74.35	-0.509	37.82
V3	433	46.36	6.24	65.27	-0.556	32.65
V4	378	41.64	9.53	72.20	-0.633	31.84

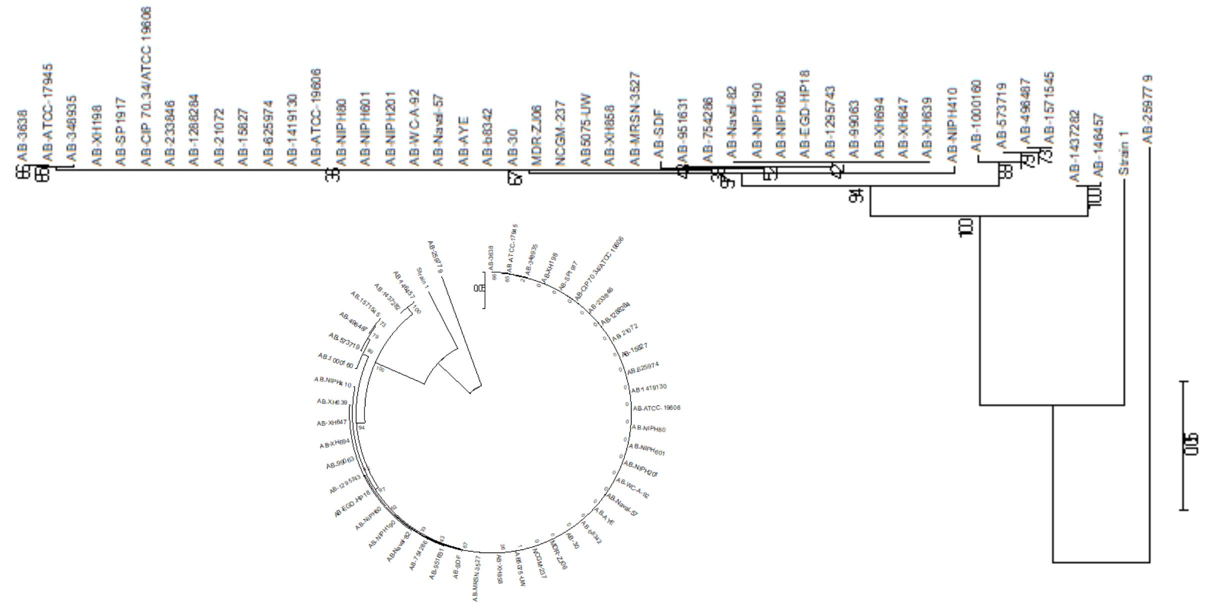

Figure SF1: 16SrRNA sequencing of all 52 strains of *Acinetobacter baumannii*. Each 52 genome sequences were searched for 16S rRNA patterns and candidate sequences were extracted. The best sequence from each genome was selected. For five genomes, no sequences were found, *A. baumannii* 118362, 855125, 940793, ABBL059 and ABBL059. The neighbour joining distance tree was made with 1000 bootstraps.

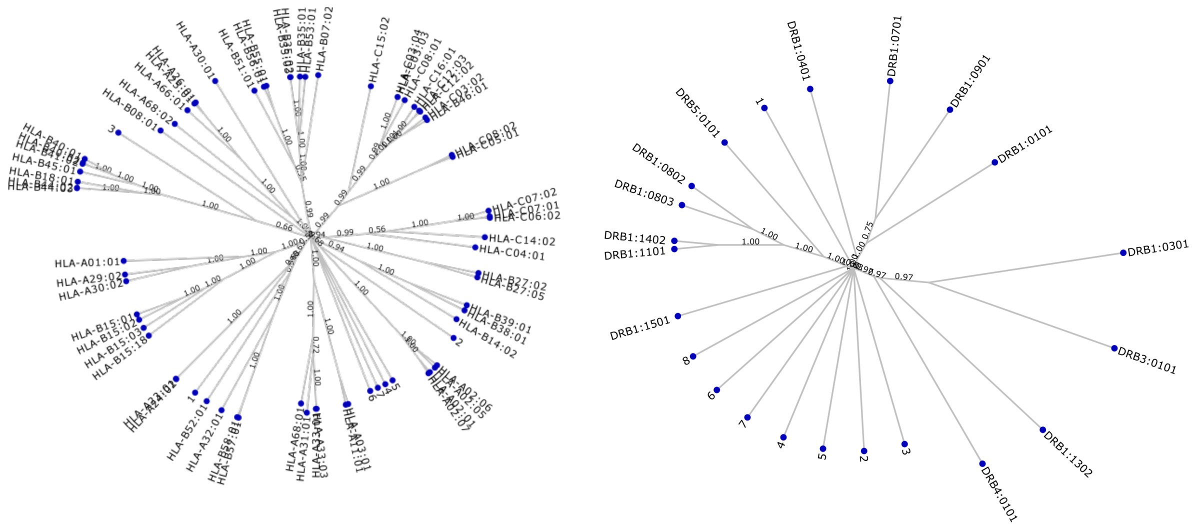

(a) (b)

Figure SF2: Graphical tree analysis of the MHC 1 (a) and MHC 2 (b) alleles.
